# The Geometry and Dynamics of Lifelogs: Discovering the Organizational Principles of Human Experience

**DOI:** 10.1371/journal.pone.0097166

**Published:** 2014-05-13

**Authors:** Vishnu Sreekumar, Simon Dennis, Isidoros Doxas, Yuwen Zhuang, Mikhail Belkin

**Affiliations:** 1 Department of Psychology, The Ohio State University, Columbus, Ohio, United States of America; 2 School of Psychology, University of Newcastle, Newcastle, NSW, Australia; 3 Center for Integrated Plasma Studies, University of Colorado, Boulder, Colorado, United States of America; 4 Department of Computer Science and Engineering, The Ohio State University, Columbus, Ohio, United States of America; University of California, Merced, United States of America

## Abstract

A correlation dimension analysis of people’s visual experiential streams captured by a smartphone shows that visual experience is two-scaled with a smaller dimension at shorter length scales than at longer length scales. The bend between the two scales is a phase transition point where the lower scale primarily captures relationships within the same context and the higher dimensional scale captures relationships between different contexts. The dimensionality estimates are confirmed using Takens’ delay embedding procedure on the image stream, while the randomly permuted stream is shown to be space-filling thereby establishing that the two-scaled structure is a consequence of the dynamics. We note that the structure of visual experience closely resembles the structure of another domain of experience: natural language discourse. The emergence of an identical structure across different domains of human experience suggests that the two-scaled geometry reflects a general organizational principle.

## Introduction

The widespread availability of sensor technology and the development of networks to accumulate information have given rise to a gold rush of applications that take advantage of our ability to track human behavior. Monitoring patient symptoms and actions as they occur provides a more relevant, sensitive and actionable approach to client care than is provided by traditional assessment methods [1], [2]. The advent of ubiquitous wearable computing devices has popularized lifelogging (i.e., tracking of personal data such as sleep, heart rate, and exercise). These technologies allow one to quantify the food consumption and physical activity patterns of individuals [3] or the dynamics of infectious disease in populations [4]. Patterns of human mobility can be analyzed for the purposes of urban planning and traffic engineering [5]. Across all of these domains, being able to rigorously quantify experience is a key enabling capability. In this paper, we focus on image data to quantify the general structure and dynamics of human experience.

Dynamical systems analysis techniques are well suited for uncovering patterns in data that are otherwise not revealed by standard statistical methods. In the current study, five participants wore a device around their necks that automatically captured images from their lives for a period of 1–2 weeks. In the following sections, we present what we believe is the first instance of application of dynamical systems techniques on lifelogging data. We use recurrence plots to visualize the pattern of recurrent visits to the same locations in visual context space over time. We then characterize the structure of experience by computing the correlation dimension of the space occupied by the images and show that visual experience has a two-scaled structure for all participants. The two scales are shown to capture different aspects of experience. In the section that follows, we demonstrate the link between the temporal sequence of visual experience and the structure of visual experience as described by the correlation dimension. To do this, we use Takens’ embedding theorem [6] to recover the dimensionality estimates directly from a series of numbers representing the time ordered images. Critically, we show that when the order of this time series of numbers is randomly permuted, the Takens procedure fails to recover the original dimensional structure. Instead, the randomly permuted time series is space filling - the recovered correlation dimension rises indefinitely as embedding dimension is increased. The Takens analysis therefore demonstrates that the dynamics of our interaction with the environment plays a key role in the structure of our experience of the world. Finally, we compare the structure of visual experience to the structure of natural language discourse [7] and suggest that the two-scaled structure may reflect a general organizational principle of human experience. We conclude by discussing potential practical applications of these results. The methods that we used to represent images and to calculate distances between them and additional methodological details about data collection and the techniques used in this paper are provided in Information S1.

## Materials and Methods

### Ethics Statement

The research protocol was reviewed and approved by the institutional review board (IRB) at the Ohio State University. Written informed consent was obtained from participants.

### Participants

Five participants provided data for this study. Participants AS and NV were recruited to collect image data using an android phone with our custom lifelogging app installed on it. Participants wore the phone around their necks in a pouch attached to a neck strap as they went about their daily lives and the app automatically captured image data. Participants were compensated at the rate of $10 per day. Additionally, three of the authors (VS, SD and YZ) also collected data. SD collected data for a period of about two weeks. Whereas SD’s android app used a movement based trigger to capture images, AS and NV used a regular interval setting (∼1 min) to trigger image captures. VS and YZ used Microsoft Research™ SenseCams to capture images at regular intervals of ∼8 seconds.

The participants had control over what data they wanted to share with the experimenters. They were instructed on how to delete data off the phone. They were also allowed to turn the app off at any time during the data collection period when they felt the need for privacy. More details about the devices and the app can be found in Information S1.

The image data sets are labeled as follows: AS (N = 2215 images, 7 days), NV (N = 2181 images, 6 days), SD (N = 4639 images, 14 days), YZSC (N = 4610 images, 7 days) and VSSC (N = 4404 images, 7 days) where “SC” in YZSC and VSSC stands for “SenseCam”. We present results for NV in the main manuscript and the analogous plots for the 4 remaining participants in the supporting materials since we get consistent results across individuals.

### The Structure of Visual Experience

#### Recurrence

Entropy calculations based on recurrence patterns in human trajectories have revealed that human mobility is surprisingly predictable [5]. Individual trajectories are characterized by a high probability of return to a small number of highly frequented locations [8]. For example, a student might have the same class at the same time on Mondays, Wednesdays and Fridays. We visualize these regularities by plotting recurrence plots [9], [10].

**Figure 1:**
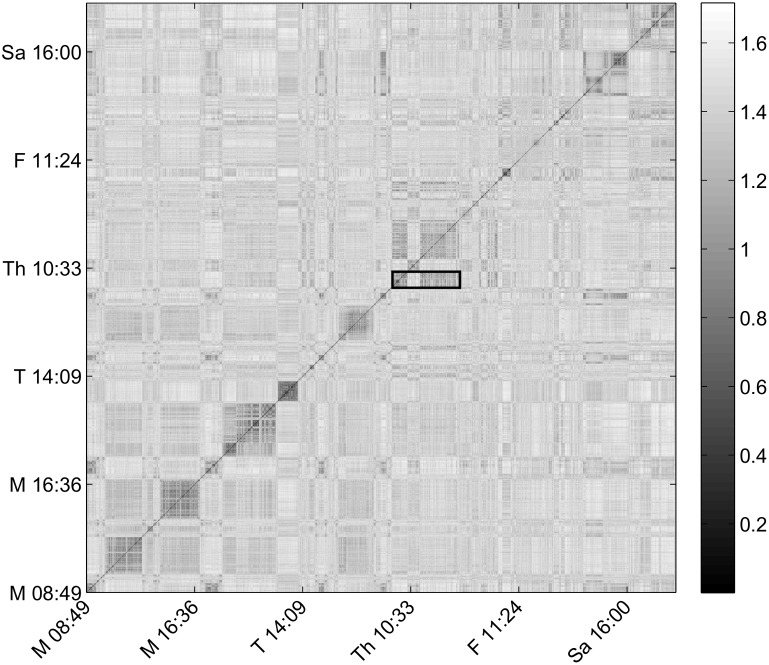
Recurrence plot for NV’s images. The images are ordered in time on both X and Y axes. The substantial dark structure around the diagonal implies that similar visual contexts were visited close in time. The off-diagonal structures represent recurrent visits to similar visual contexts at different points in time.

Figure 1 shows the unthresholded recurrence plot, sometimes also known as a global recurrence plot (RP), for participant NV. Both X and Y are time axes. The global RP is a heat map of the distance matrix. A dark point (small distance) in the RP denotes a time pair for which the dynamical system trajectory visited approximately the same region in state space (or in our case context space). Using the example given earlier, if a student has the same class at 10∶30am on Mondays, Wednesdays and Fridays, similar images would be recorded for time pairs (10∶30am Mon, 10∶30am Wed), (10∶30am Wed,10∶30am Fri) and (10∶30am Mon, 10∶30am Fri) which constitute darker points on the symmetric RP. Structures close to the diagonal of the RP represent transitions between similar spatiotemporal contexts. Since we tend to remain in the same/similar spatial context(s) contiguous in time, we expect to see many dark regions in the recurrence plot that are close to the diagonal. Off-diagonal darker structures capture returns to the same locations separated in time.

*Results and discussion.* The RPs of all 5 participants' visual experiences using three different image representations are presented in Figure S4. Figure 1 visualizes the regularity with which participant NV visited the same locations over 6 days of data collection. The pattern highlighted in the RP represents NV’s experience on Thursday. Being a graduate student, NV spent most of his day in the lab. The first dark block in the morning corresponds to NV working on his computer. Most of the images in this context block contain the computer screen. A colleague then came in to discuss research. The images in this context block capture a different view of the room that includes the other person. This context shift is denoted by the first light colored block. NV resumed working on his computer which is the second continuous dark block. Off diagonal points that lie in the same horizontal line to either side of the highlighted region represent recurrent visits to the same location (i.e., the lab) on different days. NV worked from home on Fridays and over the weekend. So there are fewer dark points in the same horizontal block to the right of the highlighted area than there are to the left. Similar characteristic regularities can be seen in the RPs of all 5 participants in Figure S4.

In the next section, we quantify the structure of experience by computing the correlation dimension of visual context space.

#### Geometry

Grassberger and Procaccia [11], [12], introduced the correlation dimension to characterize the phase space filling properties of attractors. Though there are several possible dimension measurements [13], the correlation dimension D_2_ is the most widely used due to its ease of calculation. D_2_ is a type of fractal dimension [14] because it can take on non-integer values and is related to the minimum number of variables needed to model the system's behavior in phase space [6].

To demonstrate the calculation of the correlation dimension, let us consider a thresholded recurrence plot in which images from two time points are considered recurrent and hence marked by a dark point only if the distance between the images is less than some threshold r. The number of points in the RP defined by a threshold r is the unnormalized correlation sum C(r). As we increase r, more points populate the RP and the correlation dimension D_2_ describes how C(r) scales with r (see Figure S5). For N points in an M-dimensional space, the correlation sum is given by
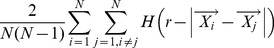
(1)where H is the Heaviside kernel function H(x) = 0 if x≤0 and H(x) = 1 if x>0. Therefore, C(r) is the number of pairs of points which are separated by less than r. For sufficiently small r and large number of points N, 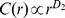. Taking logarithms of each side, we get
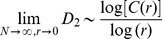
(2)


D_2_ is calculated from the slope of the straight line scaling region of a log[C(r)] versus log(r) plot. The correlation dimension is only defined for r→0 and N→∞. However, in practice, r→0 means r<<L where L is some “natural” scale of the system. So we avoid computing the slope at length scales that are comparable to the length scales of the system. Systems can exhibit different well-defined dimensions at different length scales, as long as these length scales are well separated.

**Figure 2:**
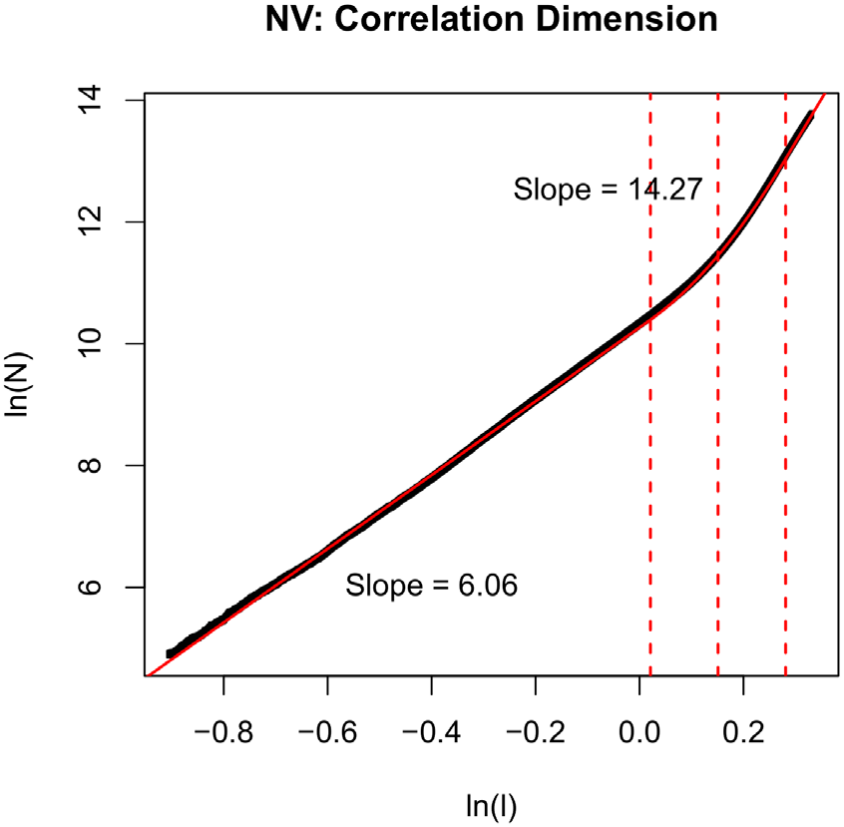
The correlation dimension plot for NV’s images shows a two-scaled geometry. The bent-cable regression lower scale correlation dimension estimate is 6.06 and the top scale correlation dimension estimate is 14.27.

*Results and discussion.* For all the analyses described in this paper, images were first converted from the RGB space into the HSV space. The images in each data set are then represented by the color correlogram [15] (see Figure S1 and Information S1 for a comparison between the color correlogram and the color histogram representations of a simple image). The justification for the choice of representation is based on the common neighbor ratio [16] (see Figure S2 and Figure S3). Following the analysis in [7], we compute the singular value decomposition (SVD) of the image by feature matrix and retain the dimensions corresponding to the top 300 singular values. The reduced image vectors are normalized and Euclidean distances are computed between pairs of these normalized image vectors. The correlation dimension plot for NV's images is shown in Figure 2.

Contexts exhibit two separate scales that are clearly distinguishable, very much like the structure of natural language discourse [7]. To fit the correlation dimension data for text corpora, Doxas et al. [7] employed the “bent-cable” regression model [17] which contains two linear segments joined smoothly by a quadratic bend (see section 3 in Information S1 for details). The quadratic segment that joins the two linear segments has a half width of γ. The two linear segments, if extrapolated, intersect at x = µ. In Figure 2, dashed vertical lines are drawn at µ and µ±γ. The use of this model to fit the correlation dimension plots helps avoid the problems associated with having to arbitrarily specify the end points of the linear regions of interest. Such arbitrary specifications can contaminate the slope estimates.

Again, following [7], we did a K-fold cross-validation (see Table S1 and section 3 in the Information S1) demonstrating that the bent cable regression model (Residual Sum of Squares (RSS) = 0.10) is superior to the linear (RSS = 4.02), second degree polynomial (RSS = 1.32) and third degree polynomial (RSS = 0.52) regression models in predictive value and generalizability. It is thereby established that there are indeed two linear regions in the correlation dimension plot. The bent-cable estimates for the lower and upper scales respectively are 6.06 and 14.27 for NV. The correlation dimension plots for the other 4 participants are presented in Figure S6. The two-scaled structure is consistent across individuals, with a lower dimension at smaller length scales and a larger dimension at longer length scales.

**Figure 3:**
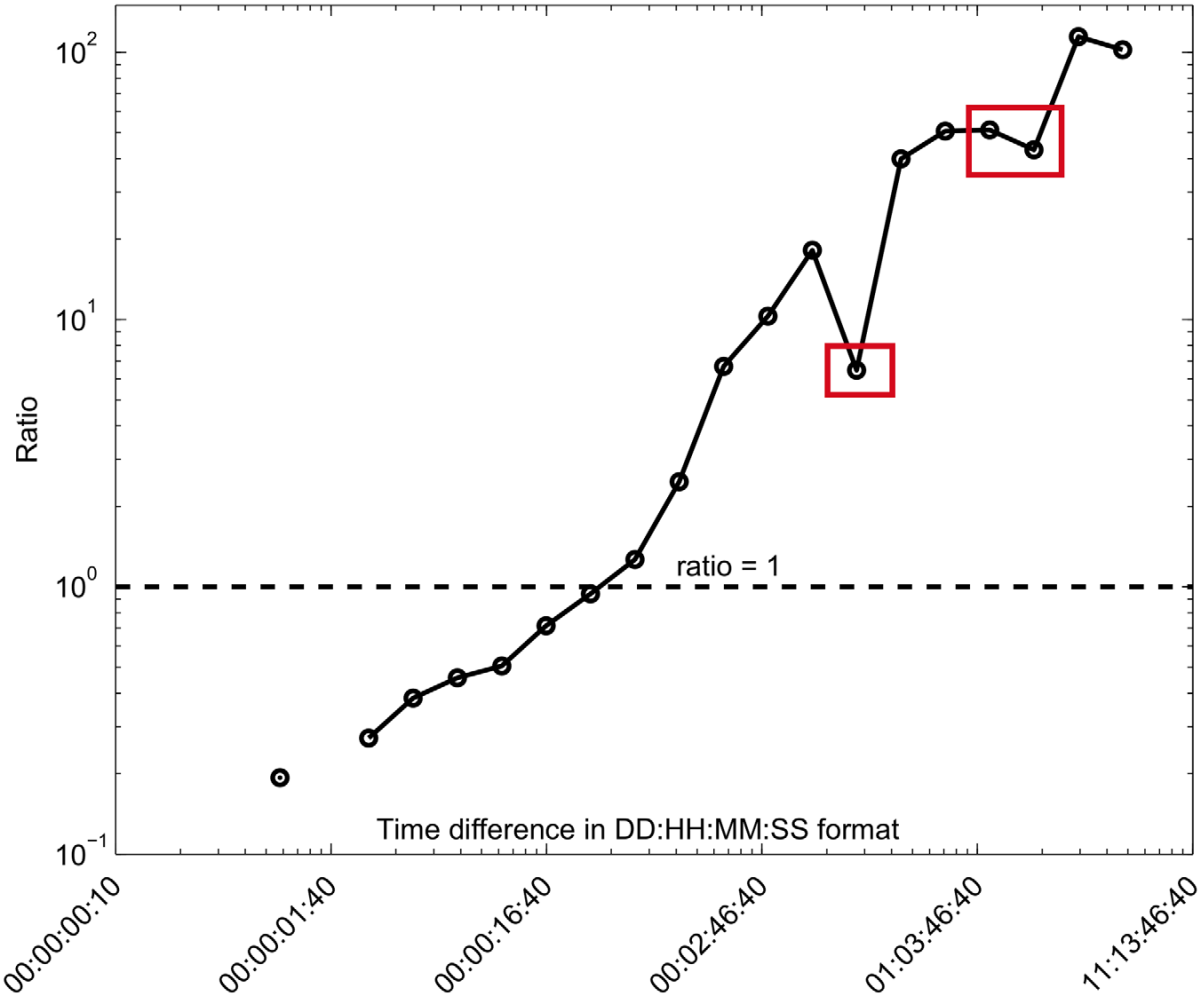
Ratio of the number of pairs of images above the bend to the number of pairs of images below the bend in NV’s correlation dimension plot as a function of binned time difference plotted on log-log axes. The ratio is approximately 1 for a time difference bin of 21 to 34 mins which lies in the range of median and mean context durations (see text for details). The drop in ratio for certain time differences, marked with the rectangles, are signatures of periodicities in the data where recurrent visits to the same context spaced by those time differences contribute more pairs to the lower scale of the correlation dimension plot thereby decreasing the ratio. Note that the second time difference bin  =  [73,118) seconds and there are no transitions with these time differences in the data - images were captured by NV’s lifelogging device at approximately equal intervals of ∼60 seconds. The minimum time interval in the data is 59 seconds followed by 60, 61, 62 and 119 seconds.

To further understand what the two separate scales mean, we calculated the ratio of the number of pairs of images above the bend to the number of pairs of images below the bend in NV's correlation dimension plot as a function of time difference. Specifically, we computed the ratio of the number of pairs of images that are separated by more than a distance of exp (µ+γ) to the number of pairs of images that are separated by less than a distance of exp (µ−γ). This ratio was plotted as a function of binned time differences (20 bins, logarithmically equally spaced) on a log-log plot for clarity. In Figure 3, the ratio is approximately 1 for a time difference bin center of 27 mins (bin = 21 to 34 mins). This means that the image pairs separated by time differences of 21–34 mins equally populate the lower and upper scales. The ratio rises above 1 for time differences longer than 34 mins, meaning that if an image pair is separated by greater than 34 mins, the pair is more likely to be part of the upper scale than the lower scale of the correlation dimension plot. The duration of a context is typically less than an hour (the mean context duration ∼50 mins and the median context duration ∼20 min for a subject pool similar to NV in lifestyle, unpublished data). Figure 3 therefore suggests that the lower scale of the correlation dimension plot primarily captures within-context transitions and the upper scale primarily captures between-context transitions.

Figure 3 also reveals signatures of periodicities in NV’s life. For example, the ratio drops for time differences around 7.7 hrs (bin = 6 to 9.7 hours, marked by the first rectangle). This was approximately the time NV spent at work every day and so it is likely that returning to the home context every day after ∼8–9 hrs contributes a significant number of pairs to the lower scale and therefore decelerates the rise of the ratio in Figure 3 for those time differences. Similarly, we see another local minimum around the bins corresponding to time differences of 1–2.7 days (marked by the second rectangle). This may be a signature of recurrence of daily/bi-daily contexts (for example, one might go back to work in 24 hour cycles, or return to a M-W-F class in 48 hour cycles).

Further insight into the nature of the two scales can be gleaned by examining the correlation dimension plots of the remaining participants. For example, the correlation dimension plot for participant AS who reported having an unusually repetitive and monotonous lifestyle during the weeks of data collection, is relatively linearized, and the dimensionalities at the two scales are closer together (lower scale dimension = 6.30 and upper scale dimension = 9.55) than the dimensionalities that characterize the other participants’ lives at the two distance scales (for example, participant SD whose lower scale dimension = 3.91 and upper scale dimension = 13.39; see Figure S6).

The bend point in the correlation dimension plot is therefore a phase transition point with the image pairs that are organized along the lower scale primarily representing within-context relationships and those above the bend capturing between-context relationships. However, the correlation dimension is a characterization of the geometry of visual experience. When describing subject NV's recurrence plot, we proposed that the off diagonal structure was a direct consequence of the sequence of context transitions he underwent. So, is the two-scaled geometry of context merely a description of the statistical distribution of visual experiences or is it linked to the specific sequence of how people transition from one context to the next? We answer this question in the next section by recovering the correlation dimension estimates from a time series of experience using Takens' delay embedding theorem [6].

### The Dynamics of Visual Experience

We described the geometry of experience as being two-scaled but is this structure related to the dynamics? Shuffling the time labels on the axes of the recurrence plots does not affect the number of points in the RP or the correlation sum C(r). This means that the correlation dimension need not be dependent on temporal properties - but rather could be strictly a property of the geometry of the points. Indeed, Doxas et al. [7] proposed a generative model that was capable of producing paragraphs of text that exhibited a two-scaled structure but the model had no temporal properties.

To establish a link between the structure and dynamics of experience, we attempt to recover the correlation dimension estimates from the temporal information in the experiential stream. We first construct a time series consisting of the values of the first dimension of the image vectors (which correspond to the largest singular values). Takens’ theorem [6] guarantees that any observable of the system, when delay embedded, will produce the same estimate for the correlation dimension. Thus, our choice of the first dimension as the observable to use in this analysis is as good as any other observable according to Takens. A moving window over the time series of this observable is then is used to construct the delay embedded vectors. Within each window, a time delay of τ is used to select values from the time series that will make up a D_e_ dimensional vector. The starting point of the window is then advanced to the next point in the time series and the procedure is repeated. The delay embedding theorem [6] ensures that the reconstruction preserves geometrical invariants like the correlation dimension, if these properties are intrinsic to the dynamics.

An appropriate value of embedding dimension D_e_ is usually chosen by computing the correlation dimension for increasing values of D_e_ until it asymptotes, at which point we assume that the system has completely “unfolded”. A more precise topological treatment of the notion of an embedding and a detailed introduction to Takens' embedding theorem are presented in section 4 in Information S1.

*Results and discussion.* The length of the time series x(t) is N = 2181 for NV’s data. Delay embedded versions of x(t) are constructed using embedding dimensions of D_e_ = {5,10,15,20,25,30,35,40,45,50} and a time delay τ that is optimized for each subject (see Figure S8 and a description of time delay selection in section 4 of Information S1).

The delay embedded vector corresponding to x(t_i_) is [x(t_i_), x(t_i+τ_), x(t_i+2τ_),…, x(t_i+(De−1)τ_)] where τ = 10 min for NV’s data. This process is repeated for i = 1 to N_max_. Beyond N_max_, we no longer have sufficient data to construct a D_e_ dimensional time delayed vector, i.e., N_max_+(D_e_−1) τ>N. For each value of embedding dimension D_e_ and time delay τ, this procedure gives us a set of N_max_ delay embedded vectors.

**Figure 4:**
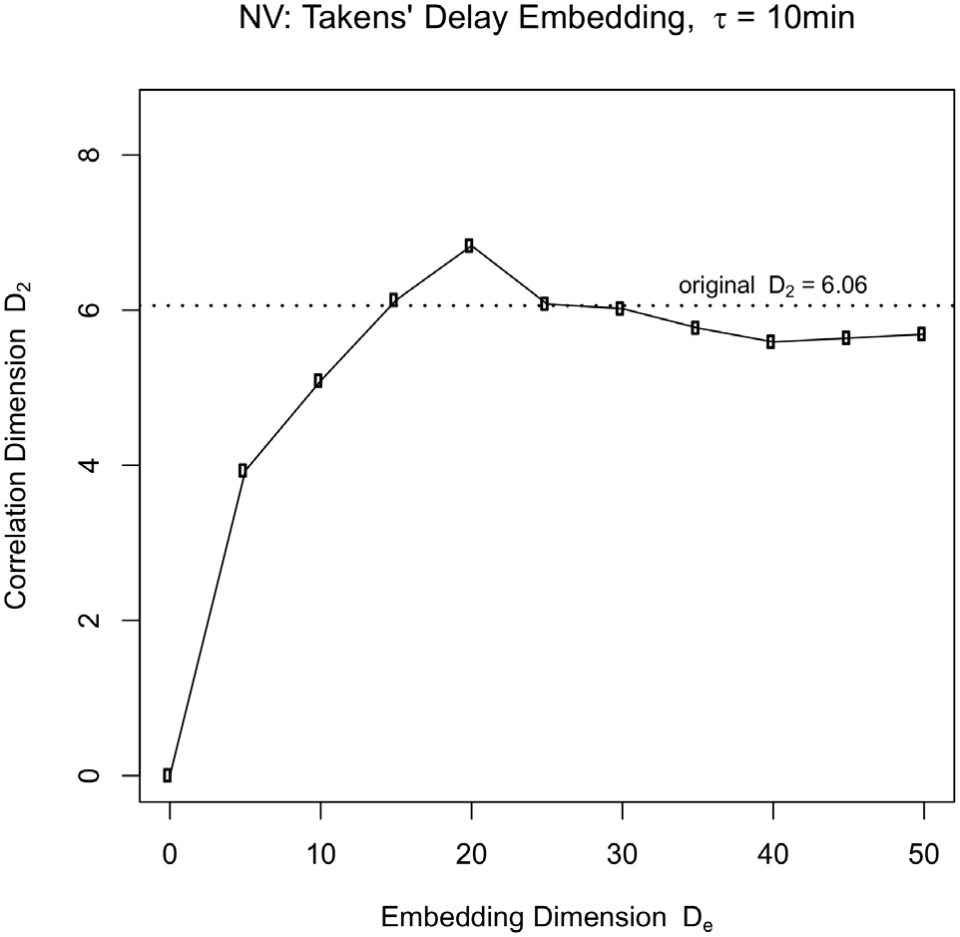
Takens’ delay embedding procedure: Recovery of the lower scale correlation dimension estimate for NV’s images. A time delay of τ = 10 minutes was used to construct the delay embedded vectors at each value of embedding dimension. As the embedding dimension is increased, the correlation dimension of the reconstructed delay embedded vectors asymptotes to the original lower scale estimate of 6.06.

Finally, we compute the Euclidean distances between the normalized delay embedded vectors and calculate the correlation dimension D_2_. Figure 4 shows that as D_e_ is increased, the reconstructed D_2_ asymptotes to the original lower scale correlation dimension estimate of NV's visual context data. We needed an embedding dimension of greater than 20 to recover the lower scale dimension of 6.06. The top scale dimension of 14.27 can in principle be recovered using higher values of embedding dimension. However, for large values of D_e_, the data are insufficient to be able to construct a sufficient number N_max_ of delayed vectors for the correlation dimension calculation.

**Figure 5:**
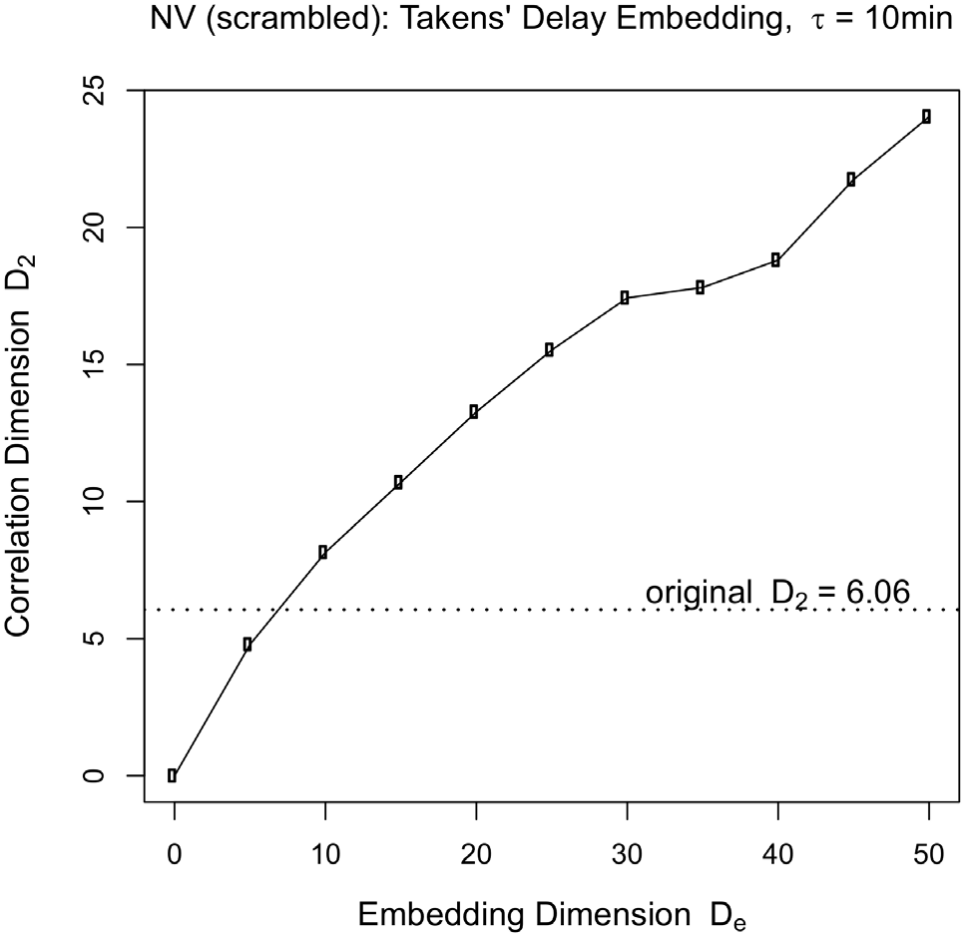
Repeat Takens’ delay embedding procedure as in Fig. 4, but with a randomly permuted time series of NV’s images. As the embedding dimension is increased, the correlation dimension of the reconstructed delay embedded vectors keeps rising and never asymptotes, demonstrating that the dimensional structure of the data is dynamic in origin.

To demonstrate that the specific order of events is necessary to be able to reconstruct the correlation dimension using the delay embedding procedure, we randomly permuted the order of NV’s image data. Figure 5 shows that it is not possible to recover the earlier structure in the absence of the correct temporal order and this result holds for any choice of τ.

The delay embedding plots for the other 4 participants are presented in Figure S7. This analysis demonstrates that how we move through our environment is linked to the two-scaled structure of visual experience.

### The Structure of Natural Language Discourse

The structure of visual experience described above resembles the structure of another domain of experience, natural language discourse. To analyze the structure of discourse, Doxas, Dennis & Oliver [7] selected five corpora in four languages: English, French, Modern and Homeric Greek, and German. These corpora included newspaper articles, texts written for children and adults, and the complete *Iliad* and *Odyssey* (Homeric Greek). Semantic spaces were constructed for each corpus using Latent Semantic Analysis (LSA; [18]). LSA is a high-dimensional model that generates representations from a corpus of natural language text that can adequately capture word-word, document-document and word-document semantic relationships [18], [19].

**Figure 6:**
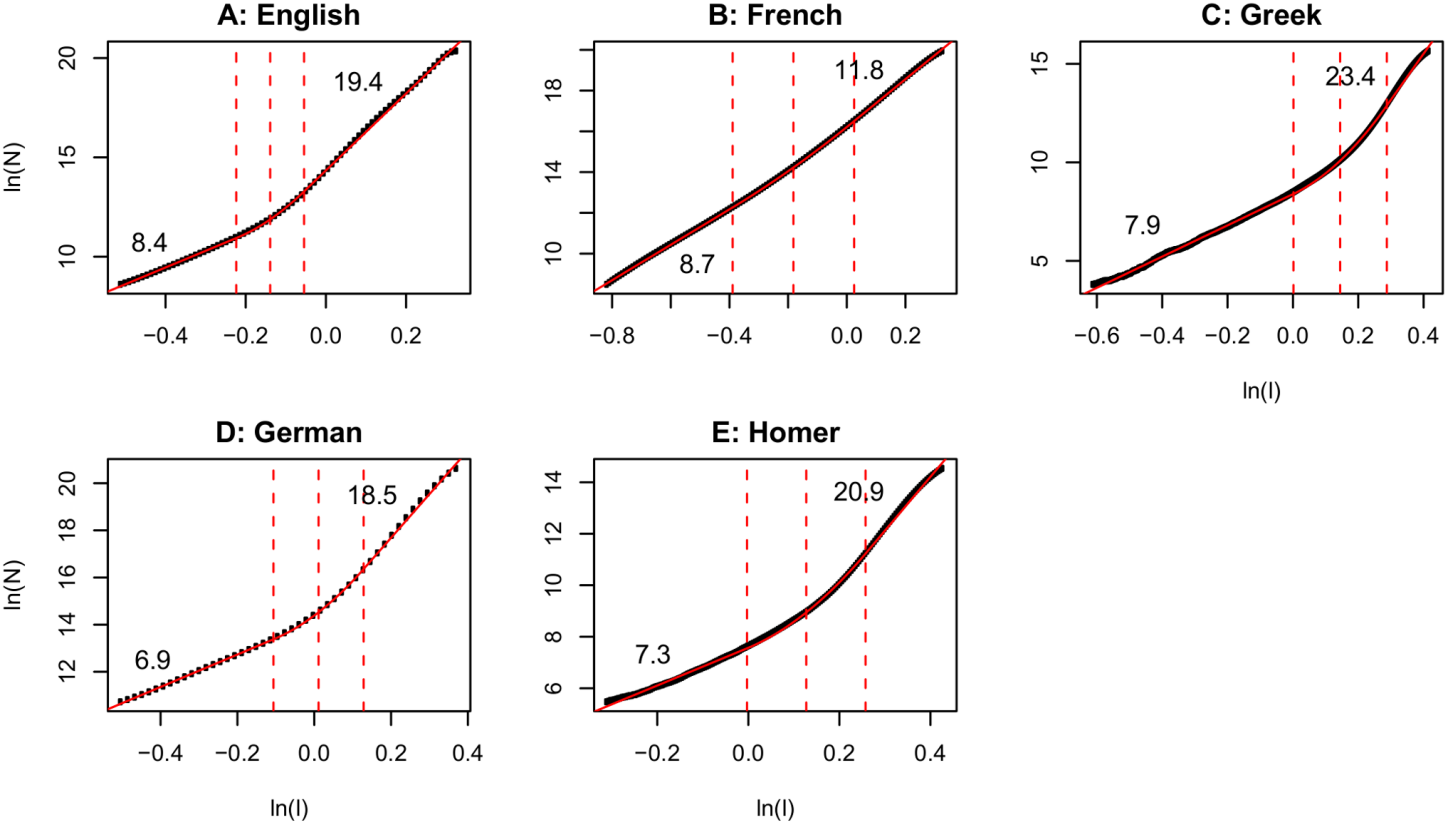
Correlation dimension of natural language discourse (reproduced with permission from Doxas, Dennis & Oliver, *PNAS*, 2010 [7]). Natural language discourse exhibits a two-scaled geometry with a smaller dimension at shorter length scales and a larger dimension at longer scales. Figure 3 and Figure S6 show that visual experience has a similar two-scaled structure.

Each paragraph in a corpus was represented as an LSA 300-dimensional vector. Euclidean distances between the LSA vector representations of paragraphs within each corpus were calculated and the intrinsic dimensionality of the semantic trajectories through each corpus was described using the correlation dimension (we followed the same analysis in this paper). Doxas et al. [7] showed that discourse trajectory has a universal two-scaled structure with the dimensionality at shorter length scales being smaller than the dimensionality at longer length scales. The overall dimensionality was also found to be surprisingly small considering that many LSA applications typically use 300 dimensions [20] to construct vector representations of documents. In the current paper, we showed that visual experience also has a similar two-scaled correlation dimension structure. The correlation dimension plots from Doxas et al. [7] have been reproduced with permission in Figure 6. Comparing Figure 6 and Figure S6 reveals the striking similarities between the structure of natural language discourse and the structure of visual experience.

Doxas et al., [7] used a version of the topics model [21] to demonstrate a generative model of prose construction that would give rise to the two-scaled structure of discourse that was observed across languages and genres. The upper scale was dominated by paragraph pairs pertaining to different topics whereas the lower scale captured relationships between paragraphs pertaining to similar topics, which agrees with our finding that the lower scale of experience captures within-context transitions and the upper scale captures between-context relationships. Though this model adequately captured the two-scaled structure of discourse, it did not have a dynamical component. In the current paper, we used Takens’ delay embedding theorem to demonstrate that the dynamics of how people move from one point to the next in the state space of the domain of experience under consideration is directly related to the two-scaled structure that is observed. Our results therefore provide an additional dynamical constraint to generative models of experience.

## Discussion

Recurrence plots of experience were presented to visualize the remarkable regularities in our movement patterns. We showed that visual context space has a two-scaled geometry with a smaller correlation dimension at shorter length scales and a larger dimension at longer length scales. The lower scale primarily captures transitions within the same context while the top scale captures transitions between different spatiotemporal contexts. Additionally, by recovering the correlation dimension estimates directly from time series that represents sequential experience, we showed that the two-scaled structure of context is related to the dynamics of how people move through their environments. The reconstruction of the structure from the dynamics, but not from a randomized sequence of images, is an important extension of earlier work that showed that the semantic space created during discourse has the same two-scaled geometry [7]. The Takens result implies that any generative account of semantic and visual experience has to not only reproduce the two-scaled geometry but must also generate specific aspects of the dynamics. Furthermore, the close correspondence in structure and the generality of these results across individuals in the case of visual experience, and languages and genres in the case of discourse, suggest that these may be universal principles that govern the workings of an interactive mind-body-environment system.

Finally, while the general properties of the dynamics of context appear common across individuals, the recurrence plots also reveal robust differences that may provide novel and useful ways of characterizing individuals. The approach taken in this paper could be put to good use in many applied settings. For example, the correlation dimension technique could potentially be used in lifelogging retrieval systems since it provides us with a personalized distance threshold for each individual that could be used for extracting images that belong to the same context as a cue image. As another example, employing a lifelogging system to track the behavior patterns of people with disorders such as schizophrenia and Alzheimer's disease may prove to be valuable in developing time critical interventions. The current study demonstrates the power of using dynamical systems methods on lifelogging data to answer complex questions about our interaction with the world.

## Supporting Information

Figure S1 **Comparison of the color histogram and color correlogram representations.** In the first image, there are 5 black pixels and 3 white pixels surrounding the pixel at the center of region marked by the grey square. In the second image, there are 3 white and 5 black pixels surrounding the same pixel. Both images contain the same total number of black and white pixels. The histogram representation being a global description of the number of pixels of each color, is identical for the two images but the correlogram representation takes into account local spatial color correlations and makes a distinction between the two images as shown by the difference in the number of pixels of j  =  {white, black} from pixel i (denoted by the arrow).
(TIF)

Figure S2 **Common neighbor ratio averaged over five subjects.** The representation with the highest common neighbor ratio is more likely than the other representations to identify images that come from the same context as being similar to each other. The correlogram representation outperforms both the color histogram and SIFT representations.
(TIF)

Figure S3 **Common neighbor ratios for individual subjects.** The representation with the highest common neighbor ratio is more likely than the other representations to identify images that come from the same context as being similar to each other. The correlogram representation outperforms both the color histogram and SIFT representations.
(TIF)

Figure S4 **Global (unthresholded) recurrence plots for 5 subjects using three different image representations.** The left panel shows the recurrence plots constructed using the color histogram representation, the middle panel using the color correlogram and the right panel using SIFT. The plots for the color histogram and correlogram representations look similar. SIFT identifies many more points as being recurrence points. Signatures of each participant’s individual lifestyles are present in their corresponding recurrence plots. AS reported having led an unusually monotonous lifestyle during the data collection period. The greater off diagonal structures in AS’ recurrence plots capture the fact that AS visited the same locations over time.
(TIF)

Figure S5 **Demonstration of the correlation dimension calculation.** **A** The thresholded recurrence plot (RP) for a threshold of r = 1.101. The number of points in this RP is C (r) and is the lower point marked in the log-log plot of panel C. **B** The RP for a threshold of r+dr = 1.290. The corresponding C (r+dr) is the upper point plotted in panel C. **C** The slope of the log[C (r)] vs log(r) plot is the estimate of the correlation dimension D_2_.
(TIF)

Figure S6 **Correlation dimension plots for individual participants.** Every single participant’s space of visual context exhibits a two-scaled geometry. The bent-cable estimates for the lower and upper scales respectively are 6.30 and 9.55 for AS, 3.91 and 13.39 for SD, 4.70 and 13.09 for YZSC, and 4.80 and 10.81 for VSSC. The solid lines indicate the best fitting bent cable regression and the dotted lines indicate the bend point and the associated width of the estimated bend.
(TIF)

Figure S7 **Takens’ delay embedding procedure: Reconstruction of the lower scale.** Different time delays were used for different participants to construct the delay embedded vectors at each value of embedding dimension. As the embedding dimension is increased, the correlation dimension of the reconstructed delay embedded vectors asymptotes to a value close to the original lower scale dimension for each participant.
(TIF)

Figure S8 **A** The average mutual information plot of NV’s time series guides our search for an optimal time delay τ for Takens’ delay embedding procedure. **B** Takens’ embedding works approximately equally well for τ = 10, 20, and 30 for NV’s data. In contrast, the randomized time series fills space, to within the limits of the number of points in the dataset, for all values of τ (only τ = 10 is presented here for clarity).
(TIF)

Table S1 **Cross validation residual sum of squares (CV RSS) for each subject and model.** Presented in the table are the mean values of CV RSS with the standard deviation presented in parentheses. The models considered are the polynomial (Poly.) regression models with degree 1 to 3, and the bent-cable regression model. The bent cable regression is chosen as the best predictive and generalizable model for every participant’s data.
(DOCX)

Information S1 **Supporting Information for “The geometry and dynamics of lifelogs: Discovering the organizational principles of human experience”.**
(DOCX)
