# Supplementary figures and images for: The Geometry and Dynamics of Lifelogs: Discovering the Organizational Principles of Human Experience
Source: PLoS One. 2014 May 13;9(5):e97166. doi: 10.1371/journal.pone.0097166 (PMC4019544; doi:10.1371/journal.pone.0097166)

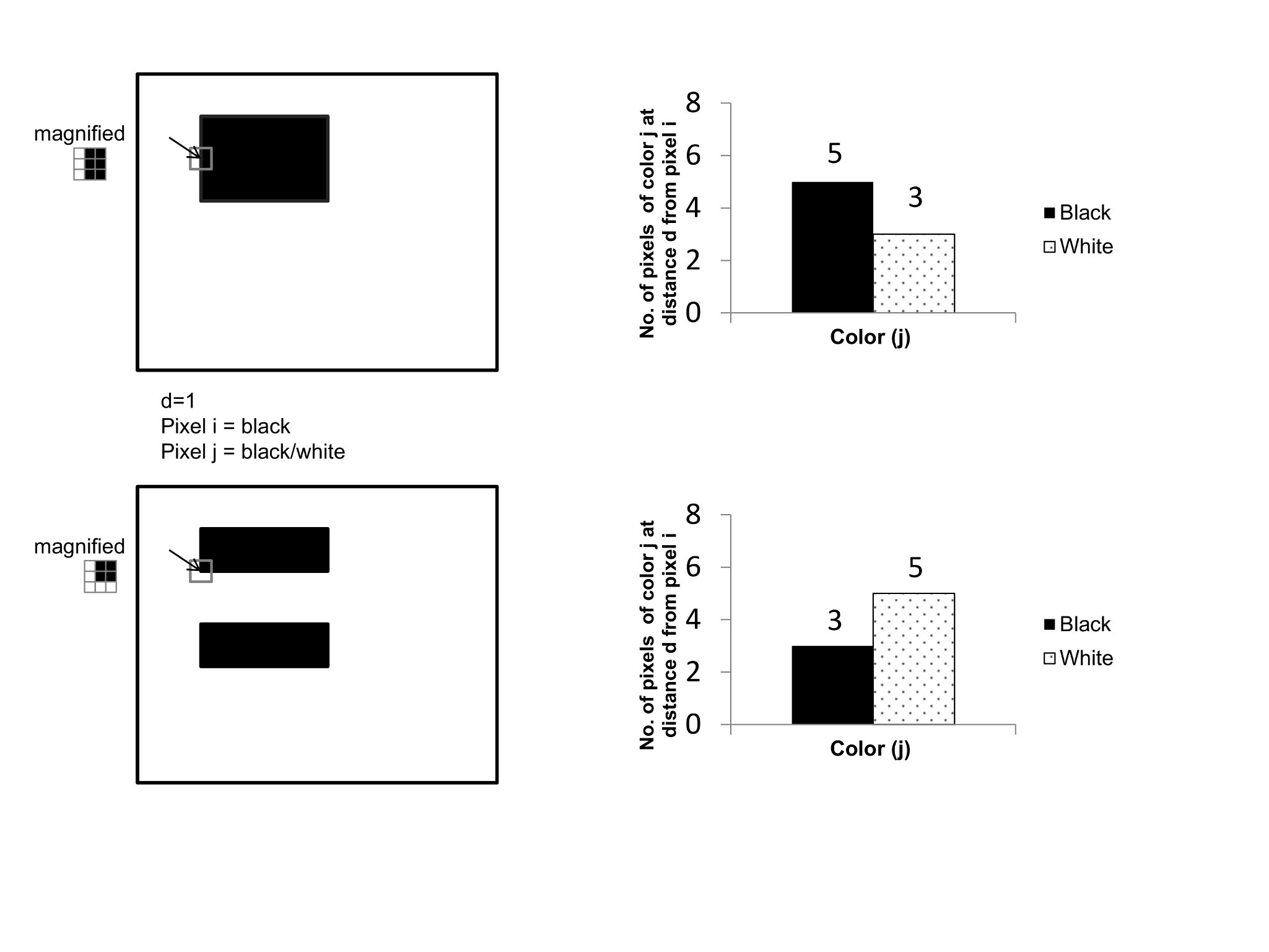

Supplement: Figure S1 — Comparison of the color histogram and color correlogram representations. In the first image, there are 5 black pixels and 3 white pixels surrounding the pixel at the center of region marked by the grey square. In the second image, there are 3 white and 5 black pixels surrounding the same pixel. Both images contain the same total number of black and white pixels. The histogram representation being a global description of the number of pixels of each color, is identical for the two images but the correlogram representation takes into account local spatial color correlations and makes a distinction between the two images as shown by the difference in the number of pixels of j = {white, black} from pixel i (denoted by the arrow). (TIF) [file pone.0097166.s001.tif]

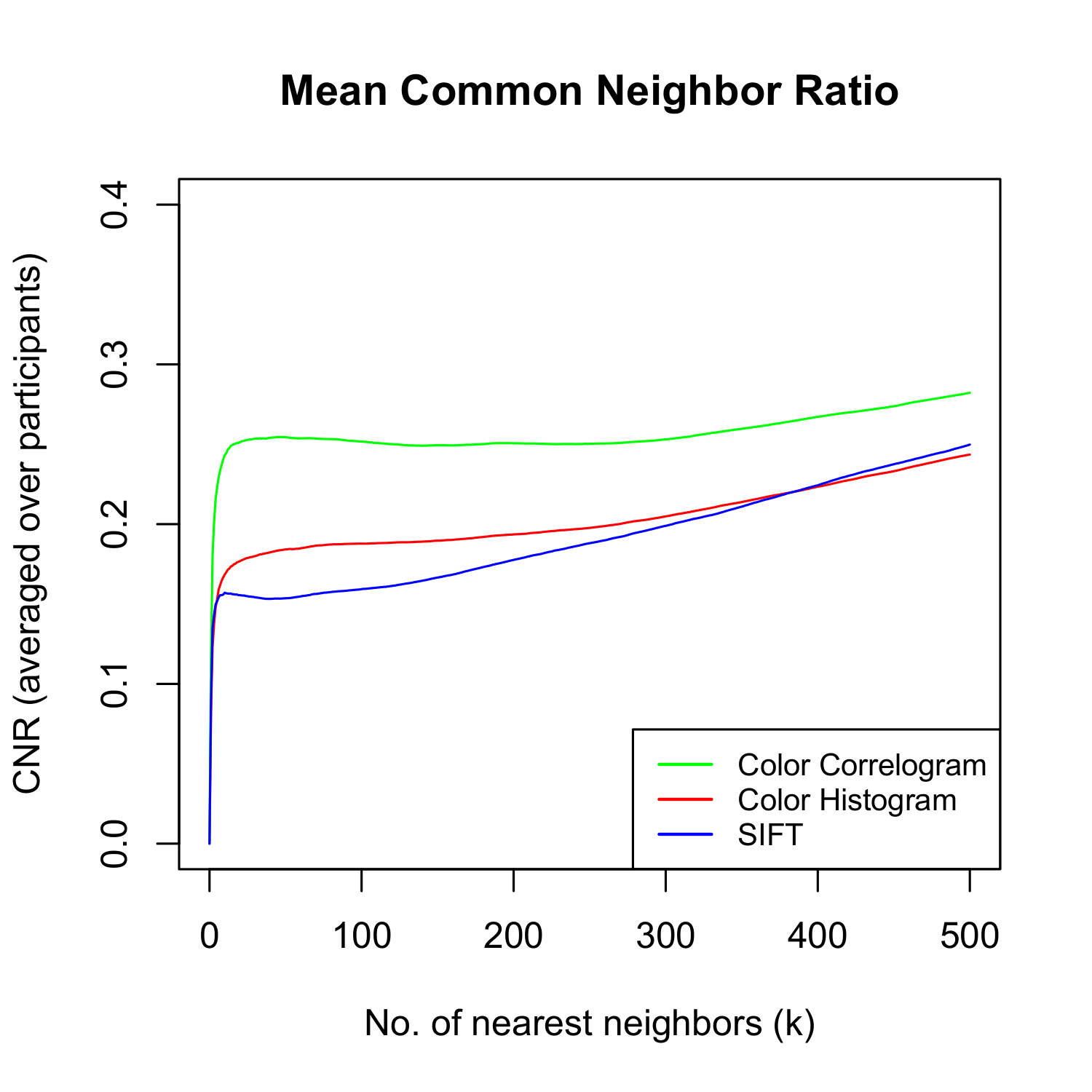

Supplement: Figure S2 — Common neighbor ratio averaged over five subjects. The representation with the highest common neighbor ratio is more likely than the other representations to identify images that come from the same context as being similar to each other. The correlogram representation outperforms both the color histogram and SIFT representations. (TIF) [file pone.0097166.s002.tif]

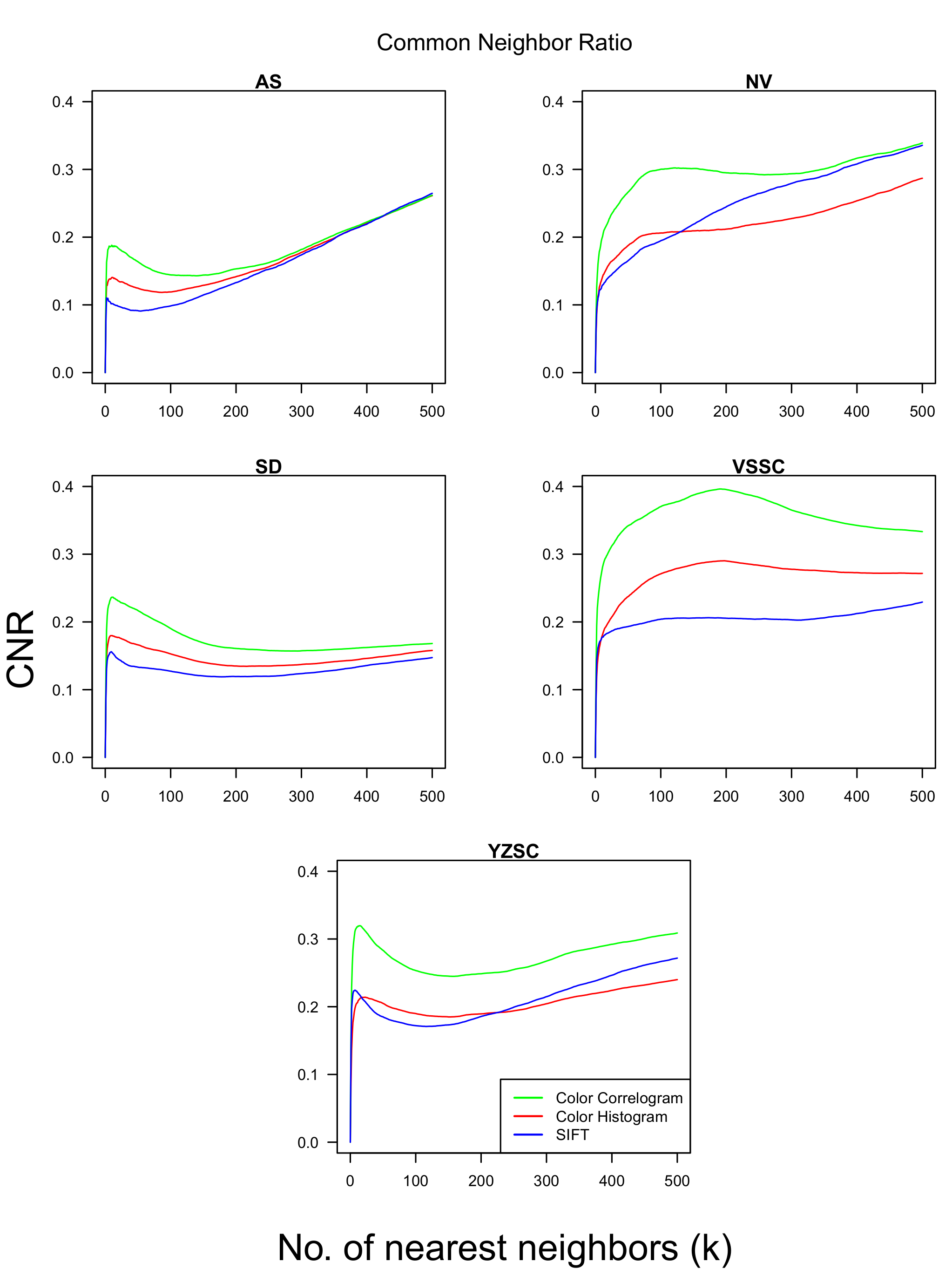

Supplement: Figure S3 — Common neighbor ratios for individual subjects. The representation with the highest common neighbor ratio is more likely than the other representations to identify images that come from the same context as being similar to each other. The correlogram representation outperforms both the color histogram and SIFT representations. (TIF) [file pone.0097166.s003.tif]

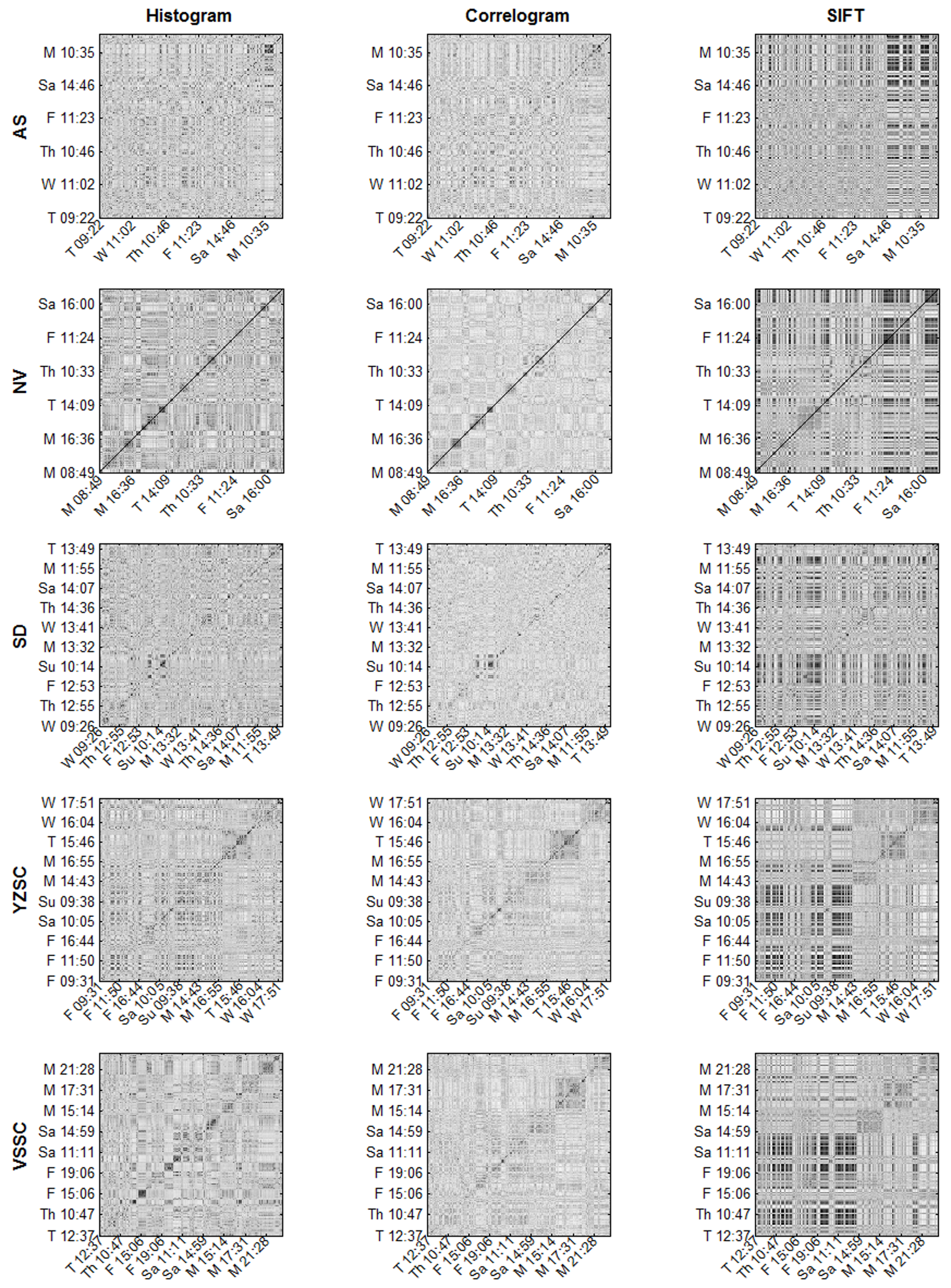

Supplement: Figure S4 — Global (unthresholded) recurrence plots for 5 subjects using three different image representations. The left panel shows the recurrence plots constructed using the color histogram representation, the middle panel using the color correlogram and the right panel using SIFT. The plots for the color histogram and correlogram representations look similar. SIFT identifies many more points as being recurrence points. Signatures of each participant’s individual lifestyles are present in their corresponding recurrence plots. AS reported having led an unusually monotonous lifestyle during the data collection period. The greater off diagonal structures in AS’ recurrence plots capture the fact that AS visited the same locations over time. (TIF) [file pone.0097166.s004.tif]

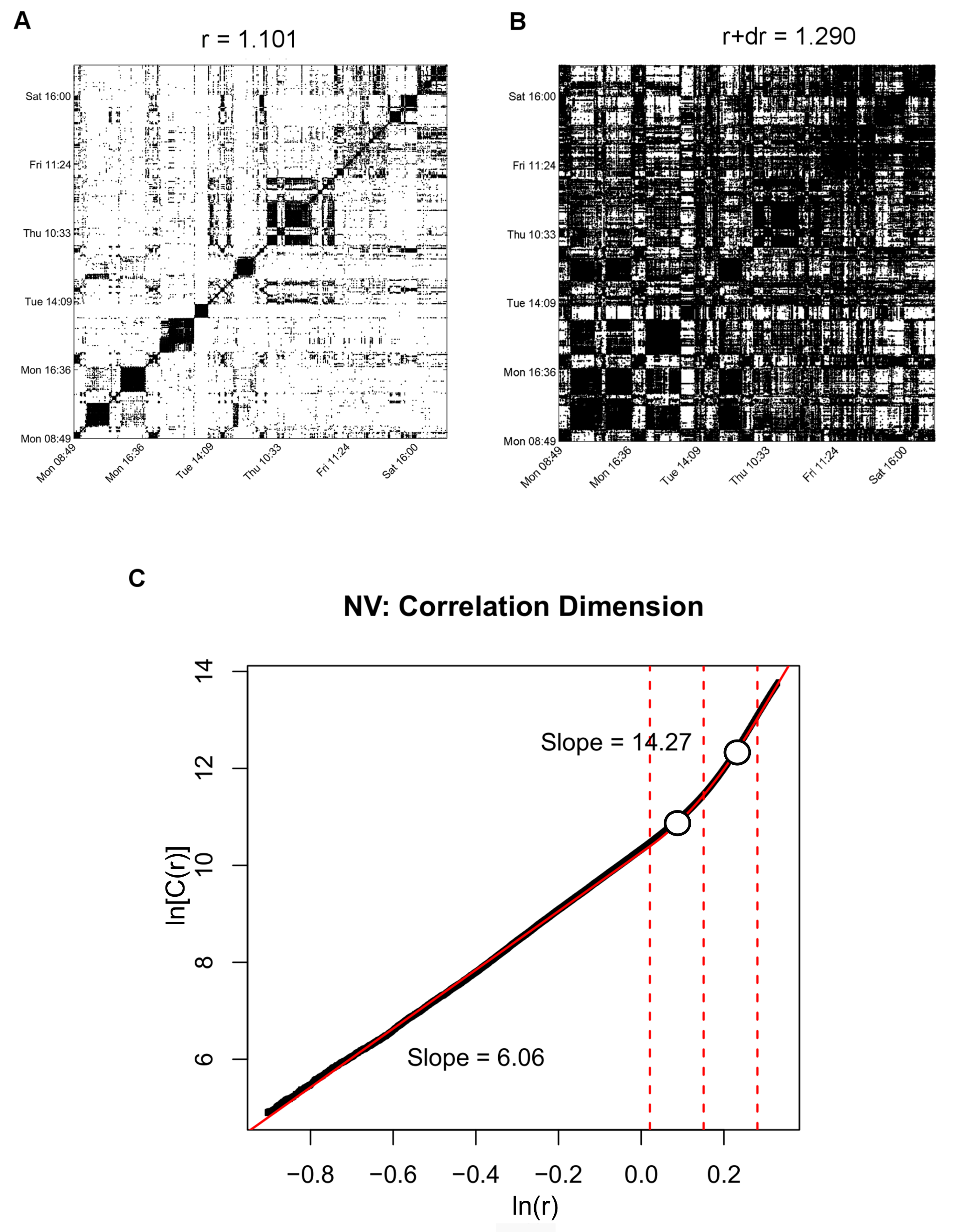

Supplement: Figure S5 — Demonstration of the correlation dimension calculation. A The thresholded recurrence plot (RP) for a threshold of r = 1.101. The number of points in this RP is C (r) and is the lower point marked in the log-log plot of panel C. B The RP for a threshold of r+dr = 1.290. The corresponding C (r+dr) is the upper point plotted in panel C. C The slope of the log[C (r)] vs log(r) plot is the estimate of the correlation dimension D2. (TIF) [file pone.0097166.s005.tif]

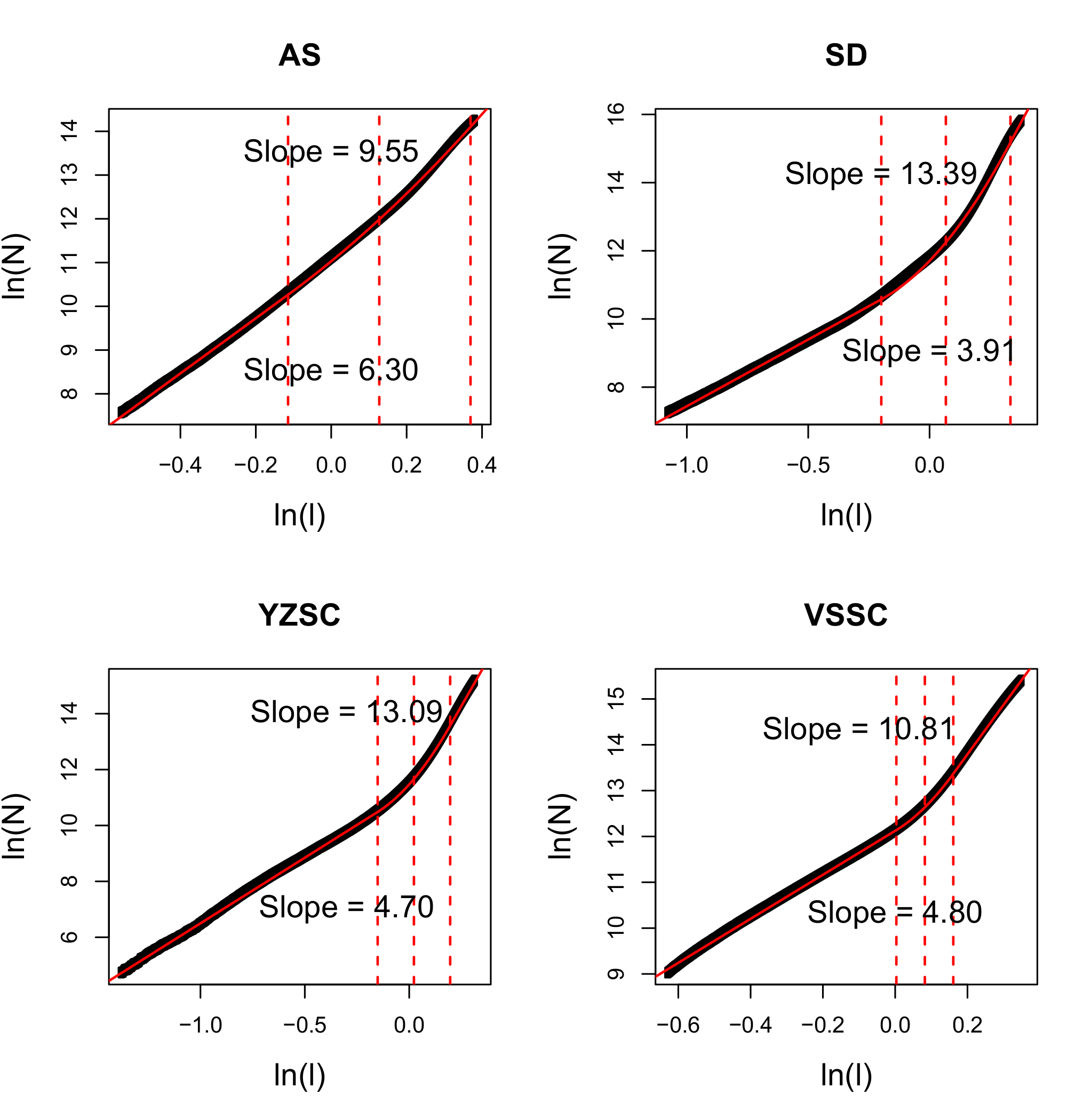

Supplement: Figure S6 — Correlation dimension plots for individual participants. Every single participant’s space of visual context exhibits a two-scaled geometry. The bent-cable estimates for the lower and upper scales respectively are 6.30 and 9.55 for AS, 3.91 and 13.39 for SD, 4.70 and 13.09 for YZSC, and 4.80 and 10.81 for VSSC. The solid lines indicate the best fitting bent cable regression and the dotted lines indicate the bend point and the associated width of the estimated bend. (TIF) [file pone.0097166.s006.tif]

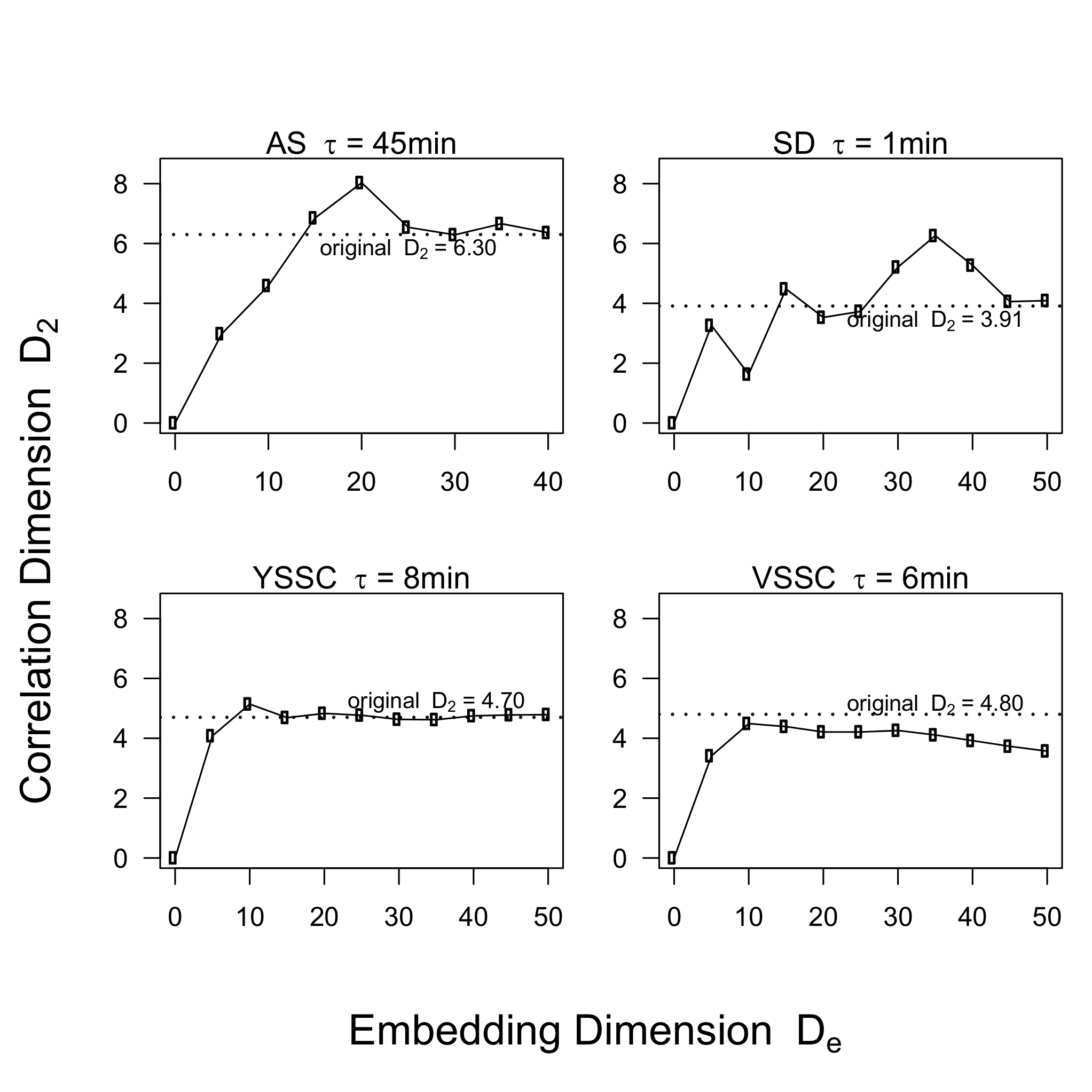

Supplement: Figure S7 — Takens’ delay embedding procedure: Reconstruction of the lower scale. Different time delays were used for different participants to construct the delay embedded vectors at each value of embedding dimension. As the embedding dimension is increased, the correlation dimension of the reconstructed delay embedded vectors asymptotes to a value close to the original lower scale dimension for each participant. (TIF) [file pone.0097166.s007.tif]

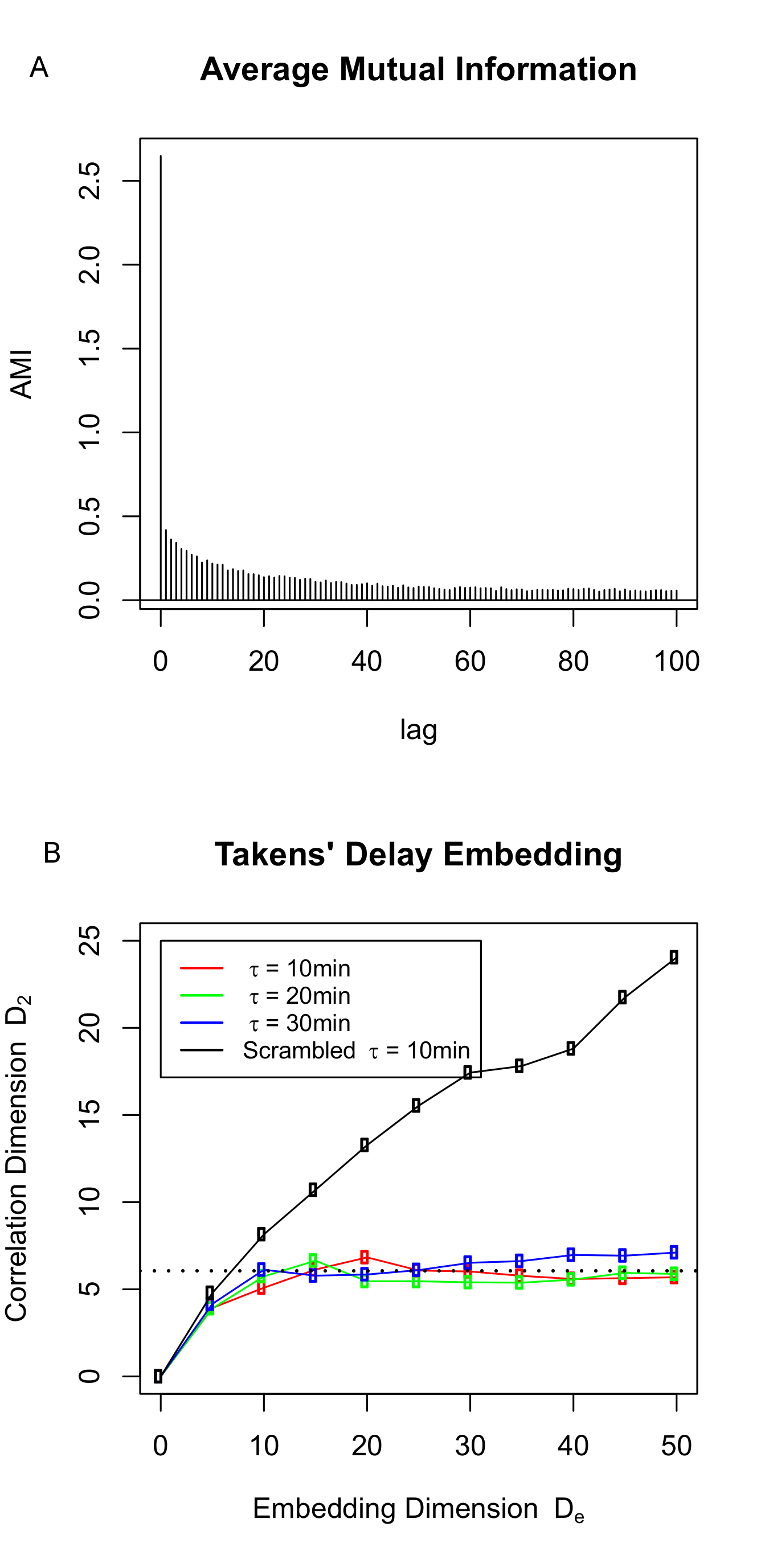

Supplement: Figure S8 — A The average mutual information plot of NV’s time series guides our search for an optimal time delay τ for Takens’ delay embedding procedure. B Takens’ embedding works approximately equally well for τ = 10, 20, and 30 for NV’s data. In contrast, the randomized time series fills space, to within the limits of the number of points in the dataset, for all values of τ (only τ = 10 is presented here for clarity). (TIF) [file pone.0097166.s008.tif]
