# Supplementary material for: The Geometry and Dynamics of Lifelogs: Discovering the Organizational Principles of Human Experience
Source: PLoS One. 2014 May 13;9(5):e97166. doi: 10.1371/journal.pone.0097166 (PMC4019544; doi:10.1371/journal.pone.0097166)
Supplement: Table S1 — Cross validation residual sum of squares (CV RSS) for each subject and model. Presented in the table are the mean values of CV RSS with the standard deviation presented in parentheses. The models considered are the polynomial (Poly.) regression models with degree 1 to 3, and the bent-cable regression model. The bent cable regression is chosen as the best predictive and generalizable model for every participant’s data. (DOCX) [file pone.0097166.s009.docx]

**Table S1.** Cross validation residual sum of squares (CV RSS) for each subject and model.

| Sub ID | Linear | Poly. 2 | Poly. 3 | Bent-cable |
| --- | --- | --- | --- | --- |
| AS | 0.78 (0.13) | 0.07 (0.02) | 0.03 (0.00) | 0.05 (0.01) |
| SD | 12.26 (4.95) | 2.34 (0.79) | 0.29 (0.07) | 0.22 (0.04) |
| YZSC | 13.80 (3.47) | 5.76 (1.05) | 1.22 (0.09) | 0.13 (0.03) |
| VSSC | 4.11 (1.17) | 1.09 (0.19) | 0.10 (0.05) | 0.04 (0.02) |
| NV | 4.02 (1.25) | 1.32 (0.34) | 0.52 (0.06) | 0.10 (0.01) |

Presented in the table are the mean values of CV RSS with the standard deviation presented in parentheses. The models considered are the polynomial (Poly.) regression models with degree 1 to 3, and the bent-cable regression model. The bent cable regression is chosen as the best predictive and generalizable model for every participant’s data.
