# Supplementary material for: The Geometry and Dynamics of Lifelogs: Discovering the Organizational Principles of Human Experience
Source: PLoS One. 2014 May 13;9(5):e97166. doi: 10.1371/journal.pone.0097166 (PMC4019544; doi:10.1371/journal.pone.0097166)
Supplement: Information S1 — Supporting Information for “The geometry and dynamics of lifelogs: Discovering the organizational principles of human experience”. (DOCX) [file pone.0097166.s010.docx]

**SUPPORTING INFORMATION S1**

Supporting Information for “The geometry and dynamics of lifelogs: Discovering the organizational principles of human experience”

Vishnu Sreekumar, Simon Dennis, Isidoros Doxas, Yuwen Zhuang, Mikhail Belkin

Contents:

Materials and Methods

Device

Representation

Section 1: Image Representations

Color Space

Color Histogram

Color Correlogram

SIFT

Choosing the best representation

Section 2: Recurrence Plots

Section 3: Correlation Dimension Plots

Bent Cable Regression

Cross Validation

Section 4: Takens’ embedding analysis plots

Supporting Information References

**Materials and Methods**

**Device**

Two different devices were used over the duration of the study to collect data. We initially used two Microsoft ResearchTMSenseCams donated to us by Microsoft to collect image data. We subsequently moved on to using our own application that we installed on android phones to collect data. This gave us more flexibility and greater control over the data.

***Microsoft ResearchTMSenseCam.*** The SenseCam has sensors which can detect changes in color, light-intensity and temperature. Changes in these sensor readings can be set to automatically trigger the SenseCam to take pictures. The camera can also be set to a timer mode where pictures can be captured periodically. Our camera captured an image once every eight to ten seconds. The camera has wide-angle (fish-eye) lens that maximizes its field-of-view thereby lending a “field perspective” to the images captured by the camera [1].

***Android Phone.*** We developed a JAVA app that acquires sensory data on Android-based smartphones. The app acquires image, time, audio (obfuscated), GPS, accelerometer and orientation information at certain time intervals which can either be fixed or be variable and determined by a movement based trigger. The movement based trigger preserves battery resource when little is changing. The app runs in the background as a service and users carried the phone in a pouch attached to a neck strap from morning till evening. The camera on the phone is given an unobstructed view by pulling back the flap of the pouch. When the phone detects WiFi and is connected to a charger, it sends the collected and stored data automatically to a remote server. This usually happens once per day at the end of the day when users charge the phone overnight. The data is sent in batch mode via SFTP protocol for added security and remains inaccessible to other users in the system.

**Representation**

For the analyses described in this paper, we needed to compute distances between images represented as vectors. From the many ways in which images can be represented, we contrasted three different images representations that are commonly used in various applications: Color Histogram [2], Color Correlogram [3], and Scale Invariant Feature Transform [4], [5]. The representations are described in section 1. We chose the color correlogram over the color histogram and SIFT representations for our image analyses based on the common neighbor ratio [6] described in section 1 (see Fig. S3 and Fig. S4).

**1. Image Representations**

We contrasted three different image representations that are commonly used in various applications: Color Histogram, Color Correlogram, and Scale Invariant Feature Transform. The color space used is described first followed by the descriptions of each image representation. Though we described the color correlogram in the main paper, we reproduce it here for completeness. Finally, the common neighbor ratio measure is introduced, on the basis of which we contrasted the representations and chose the color correlogram for the subsequent analyses.

**Color Space**

**HSV.** For many computer vision applications, the HSV (Hue, Saturation, Value) color space [7] is preferred over the better known RGB (Red, Green, Blue) color space because the HSV space keeps the color information separate from the intensity information. This makes the image representation based on HSV relatively robust to changes in appearance due to changes in lighting conditions, shadows, etc.

**Color Histogram**

For image retrieval problems, the color histogram method is a simple way of representing global image features. This representation is invariant under image rotation and translation, and has been used extensively [8]. A color histogram for an image is generated by concatenating N higher order bits for the Hue, Saturation and Value features in the HSV space. The histogram is generated by counting the number of pixels with the same color and accumulating it in 23N bins. Our representation can benefit from being less sensitive to brightness differences in order to identify similar images under different lighting conditions as similar. Quantizing the hue component more precisely than the value and saturation components makes the HSV histogram more sensitive to color differences and less sensitive to brightness and depth differences. We used a 30 x 10 x 3 hue value saturation quantization of the HSV space.

**Color Correlogram**

The color histogram has the drawback of being a purely global description of the color content in an image. It does not include any spatial information. Purely local properties when used can be extremely sensitive to appearance changes due to slight changes in angle, zoom, etc. Purely global properties like those used in the color histograms can give false positives in an image retrieval task as it tends to classify images from widely separated scenes as belonging to the same scene if they have similar color content.

A color correlogram describes global distributions of local spatial color correlations. The color correlogram γ_ci,cj_^(k)^ (I) of an image I, is a three dimensional table whose entry (c_i_,c_j_,k) is the probability of finding a pixel of color c_j_ at a distance k $\in$ {1, 2, 3, … , d} from a pixel of color c_i_ in the image. For pixels p_1_ = (x_1_,y_1_) and p_2_ = (x_2_,y_2_), we use the L_∞_ norm to measure the distance between them, such that |p_1_ – p_2_| = max{|x_1_ – x_2_|,|y_1_ – y_2_|}. Relative to the histogram, the correlogram is robust to changes in appearance caused by occlusions, zoom, and viewing angles. The size of the correlogram is *O(m^2^d)* where m is the total number of colors and i, j $\in$ {1, 2, 3, … , m}. This imposes substantial storage requirements for large values of d. So we chose to work with a compressed version of the color correlogram where we sum the conditional probabilities of color pairs over a restricted set of distances. For constructing the color correlograms, the HSV color space is quantized into 12 x 3 x 3 bins. We let k $\in$ {1, 3, 5, 7} and use a restricted version of the color correlogram as in Eq. S1.

$\bar{\gamma}_{c_{i},c_{j}}\left( I \right)= \sum_{k\in\{1,3,5,7\}} \gamma_{c_{i},c_{j}}^{\left( k \right)}(I)$ (S1)

Figure S1 demonstrates the difference between the color histogram and the color correlogram. The two images contain exactly the same number of black and white pixels. However, the first image has one black object in it whereas the second image has two distinct black objects in it. The color histogram determines that the two images are identical due to the fact that the histogram is only a raw count of the number of pixels of each color which are exactly the same for the two images. The color correlogram is sensitive to the difference between the two images due to the fact that it incorporates spatial relationships between pixels in its computation. Consider a pixel at the center of the grey square as shown in the images in Fig. S1. There are 8 pixels at an L_∞_ distance of d = 1 from it. Assume that the center pixel is black. This pixel and the pixels surrounding it are magnified to the left of each image. In the first image, there are 5 black pixels and 3 white pixels surrounding (i.e., at d = 1) the center black pixel under consideration. In the second image, this is reversed due to the change in spatial relationships between the objects in the image. We now count 3 black pixels and 5 white pixels surrounding the pixel under consideration. The color correlogram representation therefore captures the difference between these two images. The color histogram is prone to more false positives when determining which images look similar to each other.


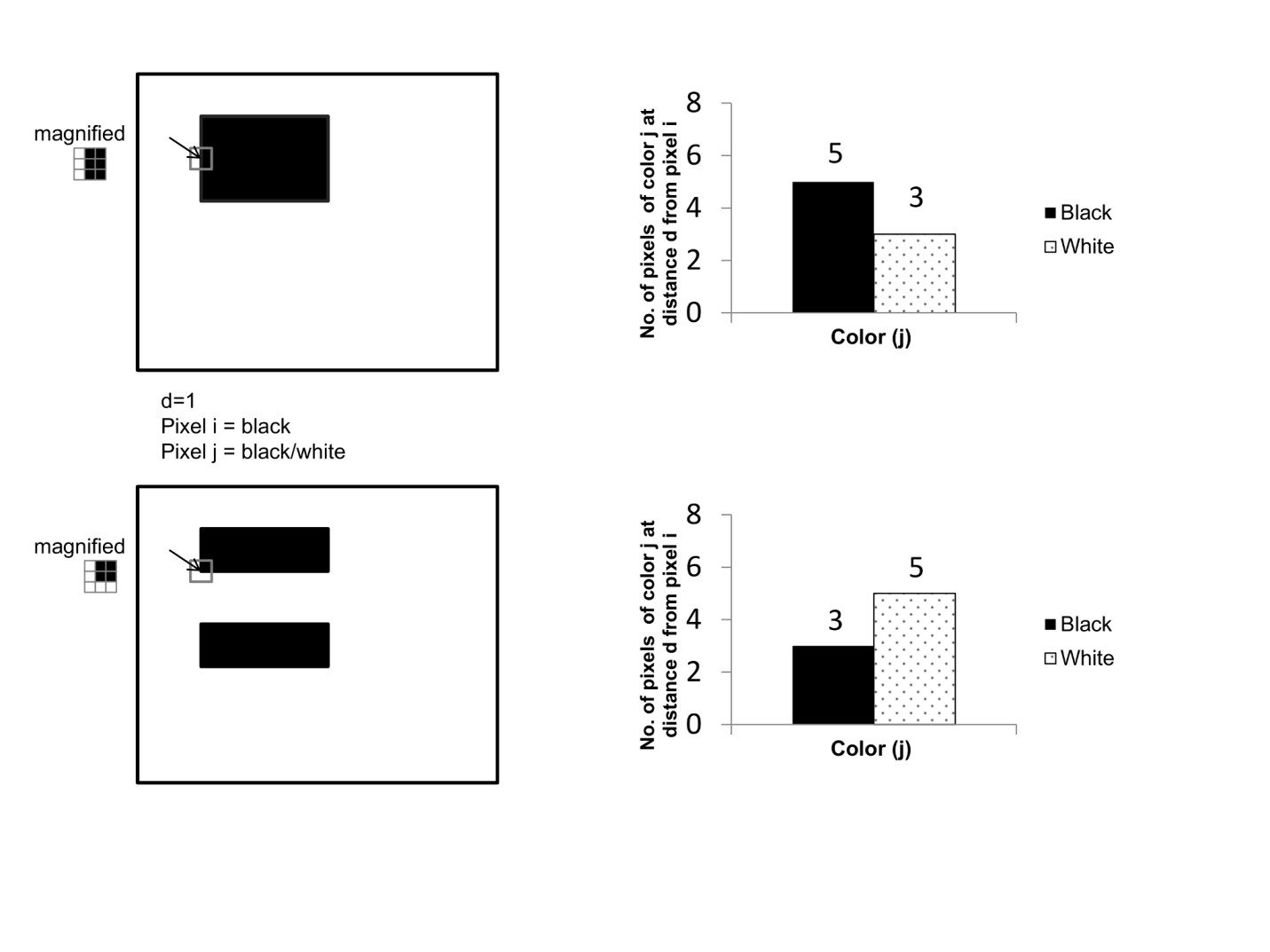


Figure S1. Comparison of the color histogram and color correlogram representations. In the first image, there are 5 black pixels and 3 white pixels surrounding the pixel at the center of region marked by the grey square. In the second image, there are 3 white and 5 black pixels surrounding the same pixel. Both images contain the same total number of black and white pixels. The histogram representation being a global description of the number of pixels of each color, is identical for the two images but the correlogram representation takes into account local spatial color correlations and makes a distinction between the two images as shown by the difference in the number of pixels of j = {white, black} from pixel i (denoted by the arrow).

**Scale Invariant Feature Transform (SIFT)**

The Scale Invariant Feature Transform (SIFT) has been used extensively with great success in computer vision, especially for object recognition. While the color histogram was a purely global description of an image and the color correlogram was a global description of local image features, SIFT features are purely local. The SIFT method represents an image as a collection of local feature vectors, each of which is invariant to image scaling, translation and rotation and partially invariant to affine transformations and changes in lighting conditions. A filtering approach is used to identify the scale and orientation invariant features.

The first step involves identifying key locations of the image that are stable across scales by looking for positions of extrema of a difference-of-Gaussian function in scale space. Given an input image I(x,y), the scale space of that image is a function L(x,y,σ) that is produced by the convolution of a Gaussian G(x,y,σ) with the image I(x,y). The difference-of-Gaussian (DoG) function convolved with the imafe, D(x,y,σ) can be used to detect stable keypoints in scale space in an efficient manner since it is easy to compute as a simple difference of images smoothed by two nearby scales [4].

$D\left( x,y,\sigma\right)=\left( G\left( x,y,k\sigma\right)- G\left( x,y,\sigma\right) \right)*I\left( x,y \right)=L\left( x,y,k\sigma\right)-L(x,y,\sigma)$ (S2)

where k is a constant multiplicative factor and * is the convolution operation in x and y. It can be shown that the DoG function is σ^2^ scale normalized if it has scaled differing by a constant multiplicative factor [4]. A scale normalized Laplacian of Gaussian function ($\sigma^{2}\nabla^{2}G$) is required for true scale invariance [9] and the DoG function with scales differing by a constant multiplicative k is a close approximation to that.

The second step is to identify the extrema of D(x,y,σ). Each sample pixel in a DoG image is compared to its 8 neighbors in the same image and 9 neighbors each in the scale above and below. This sample pixel (and the scale associated with the DoG image under consideration) is selected as an extremum of D(x,y,σ) only if it is greater than or smaller than all the 26 pixels it is compared to. This sample point is now considered a candidate SIFT keypoint. An interpolated estimate is derived for the location of the extremum by fitting a 3D quadratic function to the local sample point. The 3D quadratic function is just the Taylor expansion of D(x,y,σ) up to the quadratic terms such that the origin is at the sample point.

The third step is to reject all the candidate keypoints that are sensitive to noise in the image and retain only stable keypoints. Low contrast regions in the image are highly sensitive to noise and all interpolated extrema with pixel values below a threshold of 0.03 (pixel values $\in$ [0,1]) are discarded. Candidate keypoints may be identified along poorly localized edges since the DoG function has a strong response along edges. These are unstable to noise as well and need to be discarded. An extremum of the DoG function that lies on such an edge will have a large principal curvature across the edge and a small one in the perpendicular direction. An extremum is discarded if the ratio of principal curvatures is above a threshold (default = 10).

An orientation based on local image properties is then assigned to each keypoint. The SIFT descriptor or feature vector that is associated with any given keypoint is computed relative to the assigned orientation of that keypoint in order to achieve rotation invariance. For each keypoint, the image is first blurred using the scale of the keypoint. Local gradient magnitudes and orientations around the keypoint are calculated. Orientations are then weighted by their magnitudes as well as a Gaussian-weighted circular window with a σ that is 1.5 times the scale of the keypoint. A histogram of these weighted orientations is formed using 36 bins for 360 degrees of possible orientation values. The histogram peak represents the dominant orientation of the image near the keypoint. This orientation is assigned to the keypoint before the SIFT descriptor is computed. If there are other dominant orientations in the histogram that lie within 80% of the highest peak, new keypoints are generated with the same location and scale but with these new values of orientations.

The steps above generated keypoints each with an associated image location, scale, and orientation. The final step is to calculate the feature vector or descriptor for each keypoint with the goal of making it as invariant as possible to changes in lighting conditions and 3D viewpoint. For a given keypoint, the scale of the keypoint is used to blur the image. A 16 x 16 sample array centered at the keypoint location is considered and gradient magnitudes are computed for each pixel in this sample array. The magnitudes are weighted by a Gaussian weighting function to deemphasize the contribution of gradients that are far away from the center of the sample array. These weighted samples are then accumulated into 16 different histograms of orientations. Each of these histograms summarizes the orientation information over a 4 x 4 region of the original 16 x 16 sample array resulting in a 4 x 4 descriptor. This allows gradient samples to shift up to 4 sample positions while contributing to the same histogram in the descriptor. This makes the descriptor invariant to local positional shifts. Each histogram is made of 8 orientation bins. A 4 x 4 x 8 = 128 element feature vector is associated with each keypoint. Finally, to reduce the effects of changes in lighting conditions, the feature vector is first normalized to unit length. This serves to cancel out multiplicative changes in the feature values of a vector due to changes in image contrast. Brightness changes which have the effect of adding a constant value to image pixels do not affect the gradient values since the algorithm for computing the gradients uses pixel differences. However, some brightness changes can be non-linear when 3D structures are illuminated by different amounts in different orientations. These changes affect the magnitudes of orientations but are less likely to affect the orientations themselves. To reduce the effect of such non-linear illumination changes, the feature values in the unit vector are constrained to lie below an experimentally determined threshold of 0.2 and the vector is then renormalized to unit length. This ensures that large magnitudes of gradients have less of an influence when computing the match between images.

Since different keypoints are associated with different images, to calculate the image by feature matrix, we first did K-means on all the SIFT feature vectors in the image data set for a given subject and chose 100 cluster centers. For each image, we used these 100 centers to compute the histogram of SIFT features. The final image by feature matrix is therefore a N x 100 matrix where N is the total number of images in the data set.

***Computing distances between images.*** The images were first converted from the RGB space into the HSV space. For any given representation, the distances between images are computed in a way that makes the analysis directly comparable to the document analysis using LSA that was employed in [10]. A singular value decomposition (SVD) is carried on the image by feature matrix and the top 300 singular values are retained for the histogram and correlogram analyses. The SIFT image vectors are 100 dimensional to start with since the histogram was done on 100 centers identified by k-means. We retained the full 100 dimensions after the SVD on the SIFT image by feature matrix. The image vectors thus obtained are normalized and Euclidean distances are then computed between pairs of these normalized image vectors.

**Choosing the “best” representation:** **The common neighbor ratio**

To pick the “best” image representation for subsequent analyses, we require that our representation of choice and the associated distance measure accurately identify images from the same context as being similar to each other. Though people can and do go back to the same context at a later time, in general images that are close in time will be from the same spatial context and hence should be identified as being similar. With this in mind, we defined the common neighbor ratio (CNR) [6]. Given a positive integer k, for each image I, we find its k nearest neighbors both in the spatial domain and in the time domain. Suppose D_I_ = {I_d1_, I_d2_, … , I_dk_} are image I’s k nearest neighbors in space and T_I_ = { I_d1_, I_d2_, … , I_dk_ } are image I’s k nearest neighbors in time, then

$CNR=\frac{\sum_{I=1}^{n} \left| D_{I}\cap T_{I} \right|}{nk}$ (S3)

where n is the total number of images. If k equals n − 1 (i.e., all the other images in the set), then the ratio is 1. The method that has a higher common neighbor ratio is the better one for our purpose, which is to successfully identify images that came from the same context as similar. Figure S2 shows common neighbor ratios averaged over subjects for each image representation.

The same pattern of results holds for individual subjects and the common neighbor ratio plots for individual subjects are shown in Figure S3. We see that the correlogram representation outperforms the traditional histogram representation and the more sophisticated SIFT representation. In the next section, we will also visually compare the recurrence plots obtained using the three representations.


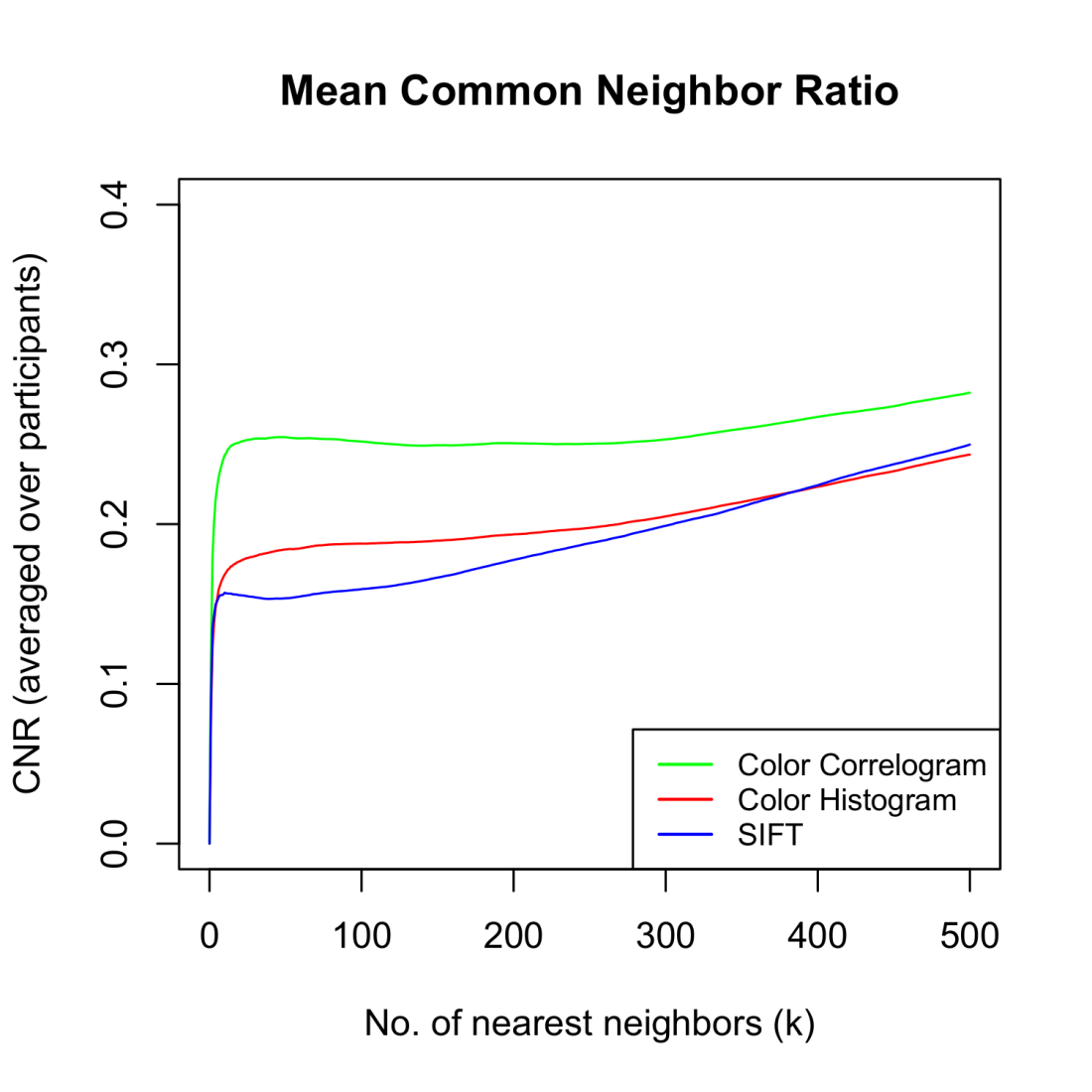


Figure S2: Common neighbor ratio averaged over five subjects. The representation with the highest common neighbor ratio is more likely than the other representations to identify images that come from the same context as being similar to each other. The correlogram representation outperforms both the color histogram and SIFT representations.


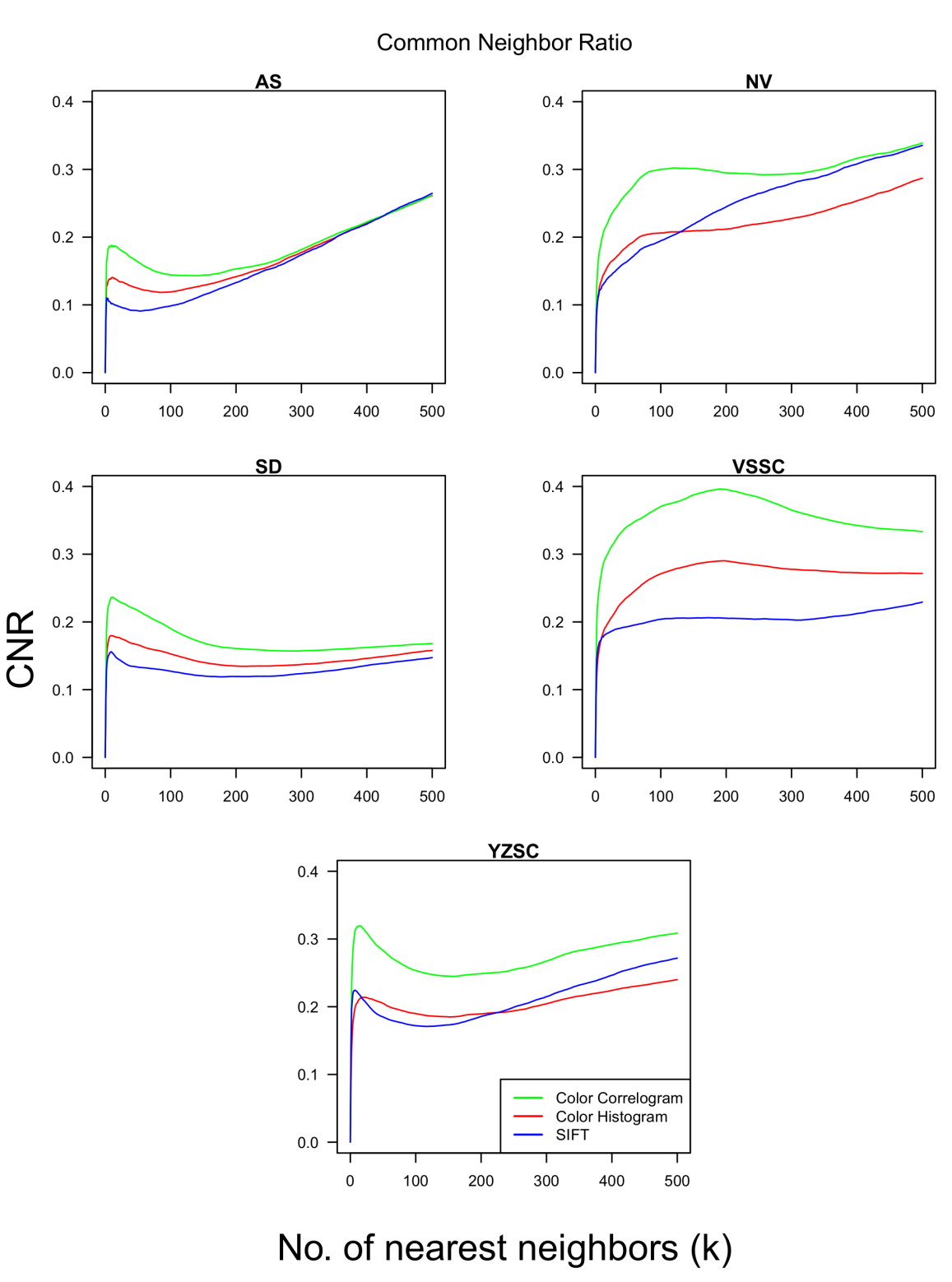


Figure S3: Common neighbor ratios for individual subjects. The representation with the highest common neighbor ratio is more likely than the other representations to identify images that come from the same context as being similar to each other. The correlogram representation outperforms both the color histogram and SIFT representations.

**2. Recurrence plots**

The recurrence plots for all 5 participants using the three image representations are presented in Figure S4. Remember that the histogram representation gives more false positives than the correlogram representation when attempting to retrieve similar images (see Figure S1 for the histogram vs correlogram comparison). So some of the space in the histogram recurrence plots gets filled in by those false positives.

The SIFT recurrence plots look suspiciously well-endowed in the off-diagonal regions. A closer look at pairs of images picked randomly from the recurrence plot revealed that SIFT identified a lot more unrelated image pairs as having come from the same context. One reason could be that color information is discarded in SIFT. It also looked like SIFT was picking lower detail images as being similar to each other. So we computed the average number of SIFT features identified in those images that were drawn randomly from the SIFT recurrence plot and compared it with the average number of SIFT features identified in the images of the entire data set. It was indeed the case that the mean number of SIFT keypoints (Mean = 23) in the images identified as highly similar by the method was much lower than the mean number of SIFT keypoints (Mean = 166) over the entire data set. When very few SIFT keypoints are identified in a lower detail image, that image is likely to have features that look similar to other low detail images with few keypoints because other more diagnostic keypoints are unavailable to make the distinction between low detail images coming from different contexts. So while SIFT might be a superior method for other computer vision applications like object recognition, it does not identify image pairs from the same spatial contexts as well as the histogram and correlogram methods.


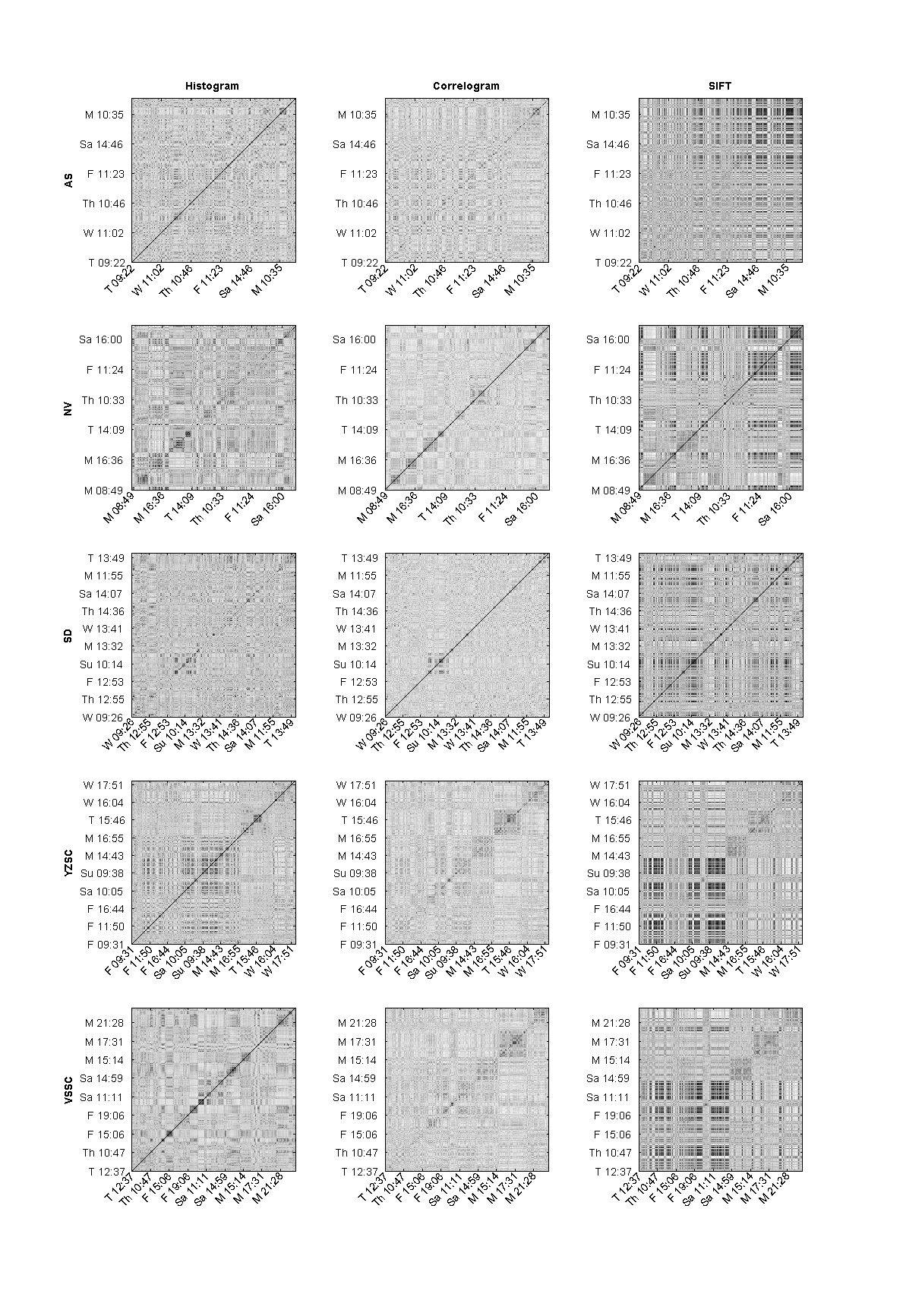


Figure S4: Global (unthresholded) recurrence plots for 5 subjects using three different image representations. The left panel shows the recurrence plots constructed using the color histogram representation, the middle panel using the color correlogram and the right panel using SIFT. The plots for the color histogram and correlogram representations look similar. SIFT identifies many more points as being recurrence points. Signatures of each participant’s individual lifestyles are present in their corresponding recurrence plots. AS reported having led an unusually monotonous lifestyle during the data collection period. The greater off diagonal structures in AS’ recurrence plots capture the fact that AS visited the same locations over time.

**3. Correlation Dimension Plots**

Figure S5 is a schematic explanation of the correlation dimension calculation. The correlation dimension measures how the correlation sum C (r) scales with r. The number of points in a thresholded version of the recurrence plot (RP) is the unnormalized C (r). The thresholded RP is plotted by identifying time pairs for which the images at those times are separated by less than some threshold distance r. As the threshold r is relaxed, more points populate the RP. The slopes of the linear regions of the C (r) vs r log-log plot is the correlation dimension estimate.

Following the analysis in [10], to fit the correlation dimension data, we did K-fold cross validation and chose the bent cable regression model [11] as the best predictive model. We present the results first followed by the descriptions of the model and the cross validation procedure in the subsequent sections.

The correlation dimension plots for individual subjects (other than NV, whose plot is presented in the main paper) are shown in Figure S6. The cross-validation results in Table S1 suggest that the bent cable regression model is superior to the polynomial regression models in predictive value and generalizability. It is thereby established that there are indeed two linear regions. The dimensionality is lower at the shorter length scales and is larger at the longer length scales, very much like the structure of natural language discourse [10]. The bent- cable estimates for the lower and upper scales respectively are 6.30 and 9.55 for AS, 3.91 and 13.39 for SD, 4.70 and 13.09 for YZSC, and 4.80 and 10.81 for VSSC.


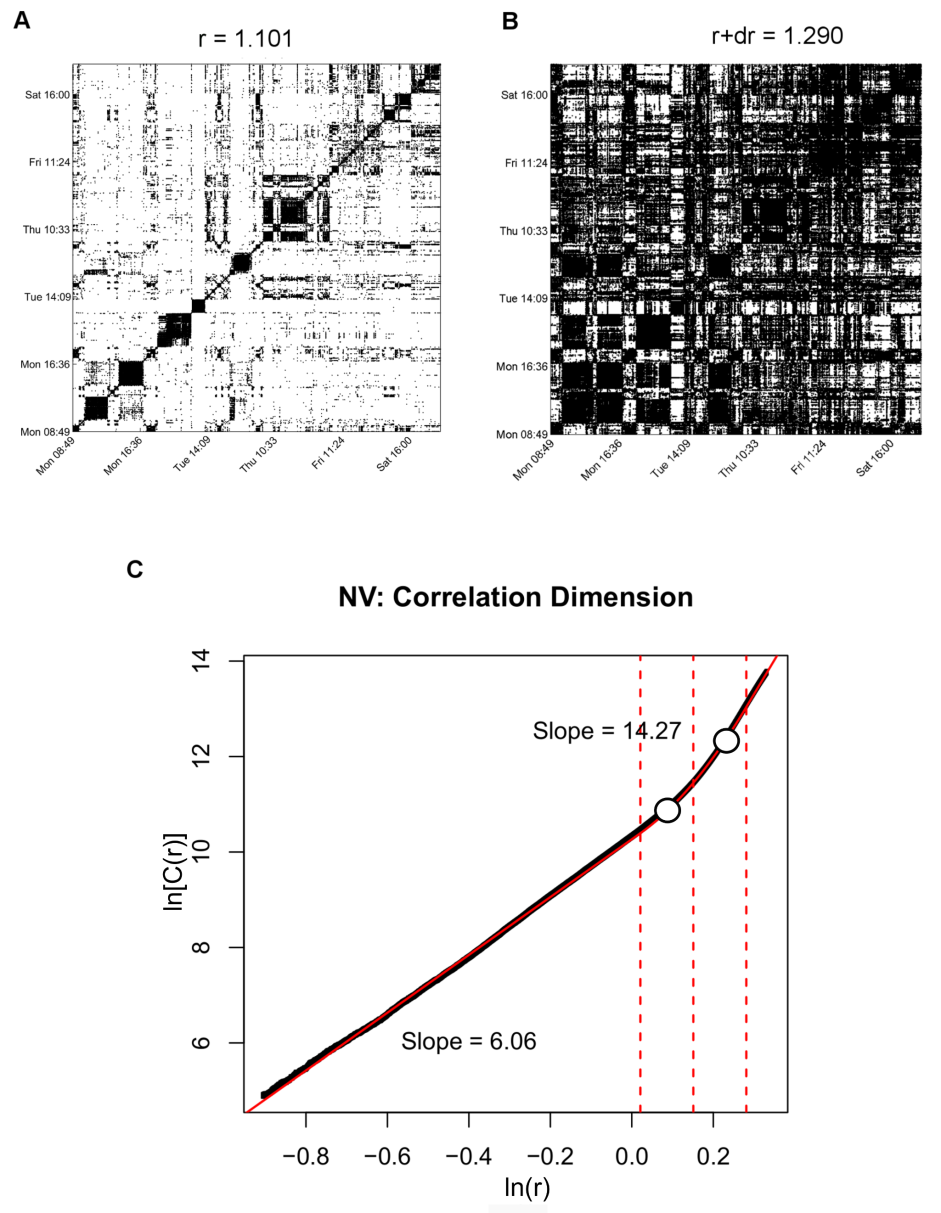


Figure S5: Demonstration of the correlation dimension calculation. **A** The thresholded recurrence plot (RP) for a threshold of r = 1.101. The number of points in this RP is C (r) and is the lower point marked in the log-log plot of panel C. **B** The RP for a threshold of r + dr = 1.290. The corresponding C (r + dr) is the upper point plotted in panel C. **C** The slope of the log[C (r)] vs log(r) plot is the estimate of the correlation dimension D_2_.


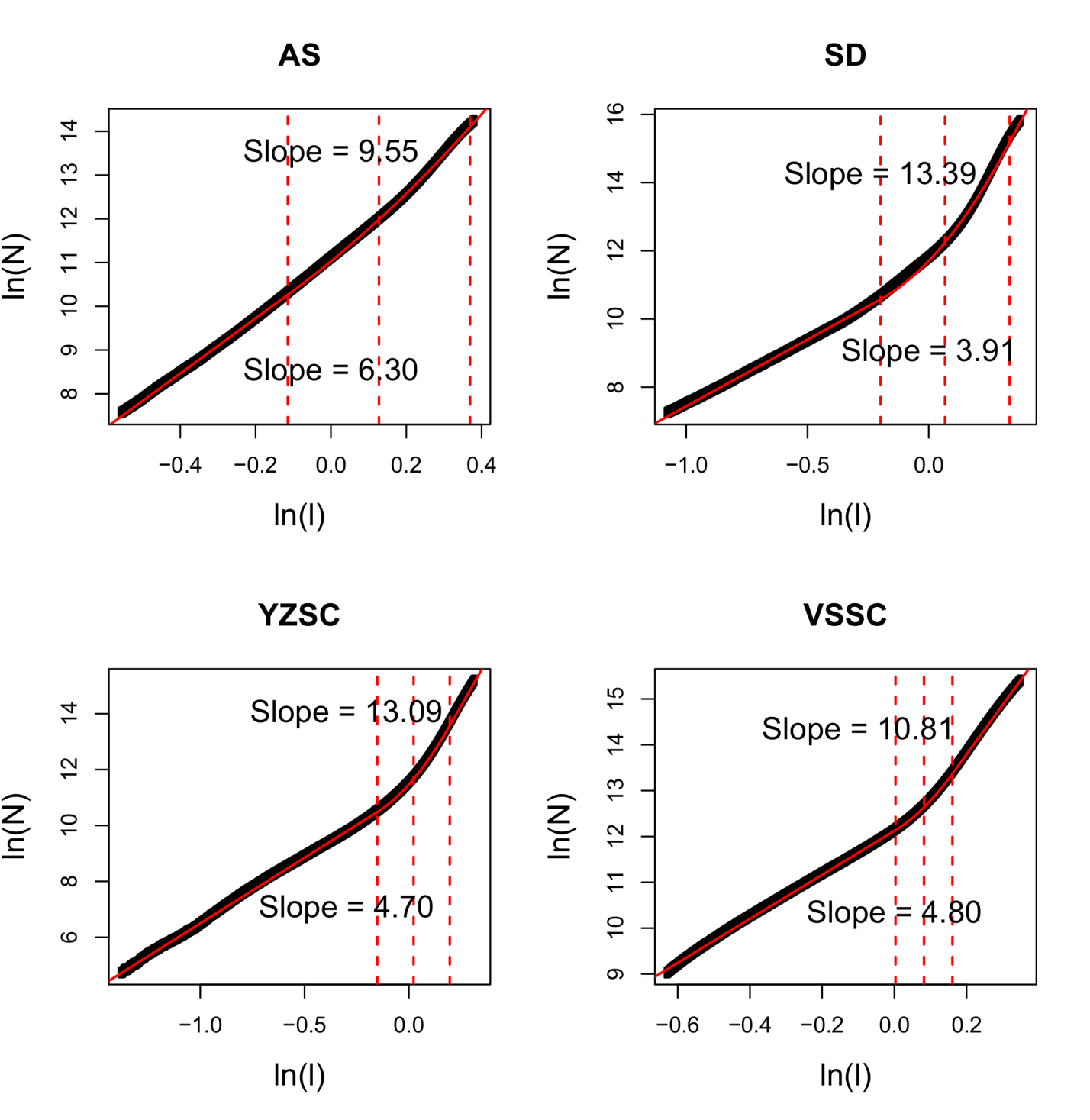


Figure S6: Correlation dimension plots for individual participants. Every single participant’s space of visual context exhibits a two-scaled geometry. The bent-cable estimates for the lower and upper scales respectively are 6.30 and 9.55 for AS, 3.91 and 13.39 for SD, 4.70 and 13.09 for YZSC, and 4.80 and 10.81 for VSSC. The solid lines indicate the best fitting bent cable regression and the dotted lines indicate the bend point and the associated width of the estimated bend.

**Bent cable regression**

The bent cable model [11] consists of two linear segments, joined smoothly by a quadratic bend. The bent cable model includes as a limiting case, the piecewise linear model with a sharp transition point, also known as the broken stick model. The bent cable regression procedure includes the estimation of the bend-width which is a measure of the abruptness of the transition. The model is described by

$f\left( t \right)= b_{0}+b_{1}t+b_{2}q(t)$ (S4)

where

$q\left( t \right)=\frac{{(t-\tau+\gamma)}^{2}}{4\gamma}(I\left\{ \left| t-\tau\right|\leq\gamma\right\}+(t-\tau)I\{t>\tau+\gamma\})$ (S5)

q(t) is the basic bent cable model which has an incoming linear phase of slope 0 and an outgoing linear phase of slope 1. The quadratic segment that joins these two linear segments has a half width of γ. The two linear segments, if extrapolated, intersect at t = τ (we used µ instead of τ in the manuscript since we later use τ to denote time delay in the Takens analysis).

In the full bent cable model f (t), the incoming lower linear phase has a slope of b_1_ and a y-intercept of b_0_ . The upper linear phase has a slope of b_1_+ b_0_ and a y-intercept of b_0_- b_2_t. The middle bend is the same as the basic bent cable q(t) but scaled by a factor b_2_. We used the “bentcableAR” R package available on CRAN to perform bent cable regression by maximum likelihood via nonlinear least-squares estimation of θ = (b_0_,b_1_,b_2_,τ,γ).

**Cross validation**

We did a K-fold cross-validation for the bent cable model and three polynomial regression models of degree 1, 2 and 3 respectively to ensure that the most generalizable model was used. The cross-validation was done by randomly dividing the data for each subject into ten equal samples. For each of these ten samples, a predictive model was formulated using the nine other samples. The predictive model was then applied on the sample that was held out. The residual sum of squares (CV RSS) for the prediction was calculated. The mean CV RSS for all the ten samples was calculated. The model with the lowest mean CV RSS value was selected as the one with the most predictive value.

Table S1: Cross validation residual sum of squares (CV RSS) for each subject and model.

| Sub ID | Linear | Poly. 2 | Poly. 3 | Bent-cable |
| --- | --- | --- | --- | --- |
| AS | 0.78 (0.13) | 0.07 (0.02) | 0.03 (0.00) | 0.05 (0.01) |
| SD | 12.26 (4.95) | 2.34 (0.79) | 0.29 (0.07) | 0.22 (0.04) |
| YZSC | 13.80 (3.47) | 5.76 (1.05) | 1.22 (0.09) | 0.13 (0.03) |
| VSSC | 4.11 (1.17) | 1.09 (0.19) | 0.10 (0.05) | 0.04 (0.02) |
| NV | 4.02 (1.25) | 1.32 (0.34) | 0.52 (0.06) | 0.10 (0.01) |

Presented in the table are the mean values of CV RSS with the standard deviation presented in parentheses. The models considered are the polynomial (Poly.) regression models with degree 1 to 3, and the bent-cable regression model. The bent cable regression is chosen as the best predictive and generalizable model for every participant’s data.

**4. Takens’ Embedding Analysis Plots**

The justification for this analysis is provided in the main text. So we will merely provide a description of Takens’ delay embedding theorem [12] and proceed to displaying the individual results.

**Takens’ delay embedding theorem**

The state space is the set of all possible states of the system; as the system evolves from some initial condition, it describes a trajectory in state space. If we obtain an adequate sampling of the state space, we can construct a model of the system. Some systems can have mutually exclusive dynamical regimes that have to be sampled separately and so we might only be able to reconstruct features of the dynamical regime of the system that has been sampled. Furthermore, noise and finite sample sizes can also limit the features of the system that can be discerned and modeled, even within the dynamical regime that our data samples.

Let us assume that Φ is a smooth dynamical system in Euclidean space R^k^ such that s(t) = Φ^t^(s(0)) where s(t) is the state of the system at time t. The data that is typically available to us is x(t) $\in$ R which we assume is obtained by some measurement function h(.) acting on s(t) such that x(t) = h(s(t)).

The first step in analyzing such a time series x(t) in terms of dynamical systems theory is to reconstruct the original state space. Since we typically do not have access to Φ and h, it is not possible to reconstruct the original space precisely but instead we aim to reconstruct a space that is “equivalent” to the original state space in some sense so that analyses of geometrical and dynamical properties of the system can be carried out in this new space. In other words, we need to reconstruct an embedding of the original state space based on the time series information (x(t)) that we do have access to. Takens’ Delay Embedding Theorem [12] guarantees that an embedding can be constructed based on x(t) if we have an infinitely long record of noise-free data. To intuitively understand the theorem, we need to first define what an embedding is. Informally, an embedding is a map from the original state space to another space where distinct points and distinct tangent directions of the original space are not collapsed, so as to preserve the topological and dynamical properties of the original space.

An accurate definition of embedding depends on differential structure [13]. Let us assume that we are interested in the long term dynamics of a system whose trajectories are asymptotic to an attractor A. Since tangent spaces are well defined only on compact sets, let us further assume that A is a compact subset of Euclidean space R^k^. Now, our goal is to find a smooth map F that maps R^k^ to another Euclidean space R^n^ such that A is embedded in R^n^ by F(A). In formal topological language, a map F is said to be an embedding of a compact set A if and only if F is a smooth diffeomorphism from A onto its image F(A) and is additionally also an immersion. A diffeomorphism is a smooth one-to-one map with a smooth inverse. F must be one-to-one at every point *s* on A so as to not collapse two different points. Differential structure is preserved only if F is an immersion, i.e., the derivative map DF(*s*) is also one-to-one at every point *s* on A. So this second condition ensures that tangent directions are not collapsed by the map.

The Whitney Embedding Theorem [14] states that a generic map from a d-dimensional manifold to a 2d+1 dimensional Euclidean space is an embedding (as defined above). Since 2d+1 independent and simultaneous measurements of the system can be considered a map of the system to the 2d+1 dimensional space, vectors of 2d+1 independent measurements of the system can be used to reconstruct the original state space. As mentioned at the outset however, what we typically have access to is not 2d+1 independent signals measured from the system but one single signal, a time series x(t) (e.g. EEG recordings, time series of stock returns). Even if we did have access to simultaneous measurements on the system, we would then have to ensure that those are truly independent measurements. An alternative reconstruction method that does not require us to have 2d + 1 independent measurements of the system is the method of derivatives that was investigated numerically by Packard, Crutchfield, Farmer & Shaw [15]. They sampled one coordinate of a well-known 3D chaotic dynamical system, the Rössler system, to get a time series x(t). The set of vectors y(t), constructed by taking successively higher order derivatives of the same signal x(t) was numerically shown to mimic the behavior of the system in the original state space and was shown to preserve geometrical invariants like the Lyapunov exponent.

$y\left( t \right)={(x\left( t \right),\hat{x}^{'}\left( t \right), \ldots, \hat{x}^{\left( m-1 \right)}(t) )}^{\dagger}$ (S6)

where $\hat{x}^{j}$ is a numerical approximation to the j^th^ derivative of x(t). To provide an intuitive motivation for Takens’ Delay Embedding Theorem which we will state next, it would be useful to note that for a small time difference τ , we can approximate the time derivatives in Eq S6 in terms of delay coordinates $(x\left( t \right), \frac{x\left( t \right)-x(t-\tau)}{\tau}, \frac{x\left( t \right)-2x\left( t-\tau\right)+x(t-2\tau)}{\tau}, \ldots)$.

We are now ready to formally state the delay embedding theorem proposed by Takens. Takens investigated the delay reconstruction map F with time delay τ and embedding dimension m.

$$F\left( s\left( t \right) \right)=\left( h\left( s\left( t-\left( m-1 \right)\tau\right) \right), h\left( t-\left( m-2 \right)\tau\right), \ldots, h\left( s\left( t \right) \right) \right)^{\dagger}$$

$=\left( x\left( t-\left( m-1 \right)\tau\right), x\left( t-\left( m-2 \right)\tau\right), \ldots, x(t) \right)^{\dagger}$ (S7)

where h is an observation function acting on the attractor states s and produces a time series x(t) = h(s(t)).

Takens’ Delay Embedding Theorem states that the delay maps of dimension m = 2d+1 that form an embedding of a compact manifold of dimension d are dense, provided that the measurement function h : R^d^🡪R is C2. The measurement function must also couple all the degrees of freedom of the original space so that no information is lost in the embedding. In other words, the measurement function must satisfy certain genericity conditions. The theorem allows the use of almost any value of time delay τ but it restricts the use of certain values of τ in order to satisfy the aforementioned genericity conditions so that self-crossings of trajectories in the reconstructed space are not permitted. Self-crossings would violate the condition that the map F be one-to-one. Takens further proved that the delay embedding reconstruction preserves geometrical invariants like the correlation dimension. A detailed topological proof can be found in [12]. The theorems mentioned so far apply to smooth manifold attractors. Sauer, Yorke & Casdagli [13] extended these results and showed that an attractor (which can be fractal) of box-counting dimension d can be reconstructed with m time delayed coordinates constructed from one generic observation where m is an integer greater than 2d. The correlation dimension D2 is a lower bound on the box-counting dimension [16] but in many practical cases, they are identical.

All the subjects, except SD, captured images using devices that triggered image captures at regular intervals. For SD’s data, we first prepared a time series x(t) that is approximately equidistant in time by using the image time stamps. We carried out the rest of the analysis as described in the main text on this time series. The Takens embedding plots for the 4 participants other than NV are displayed in Figure S7.
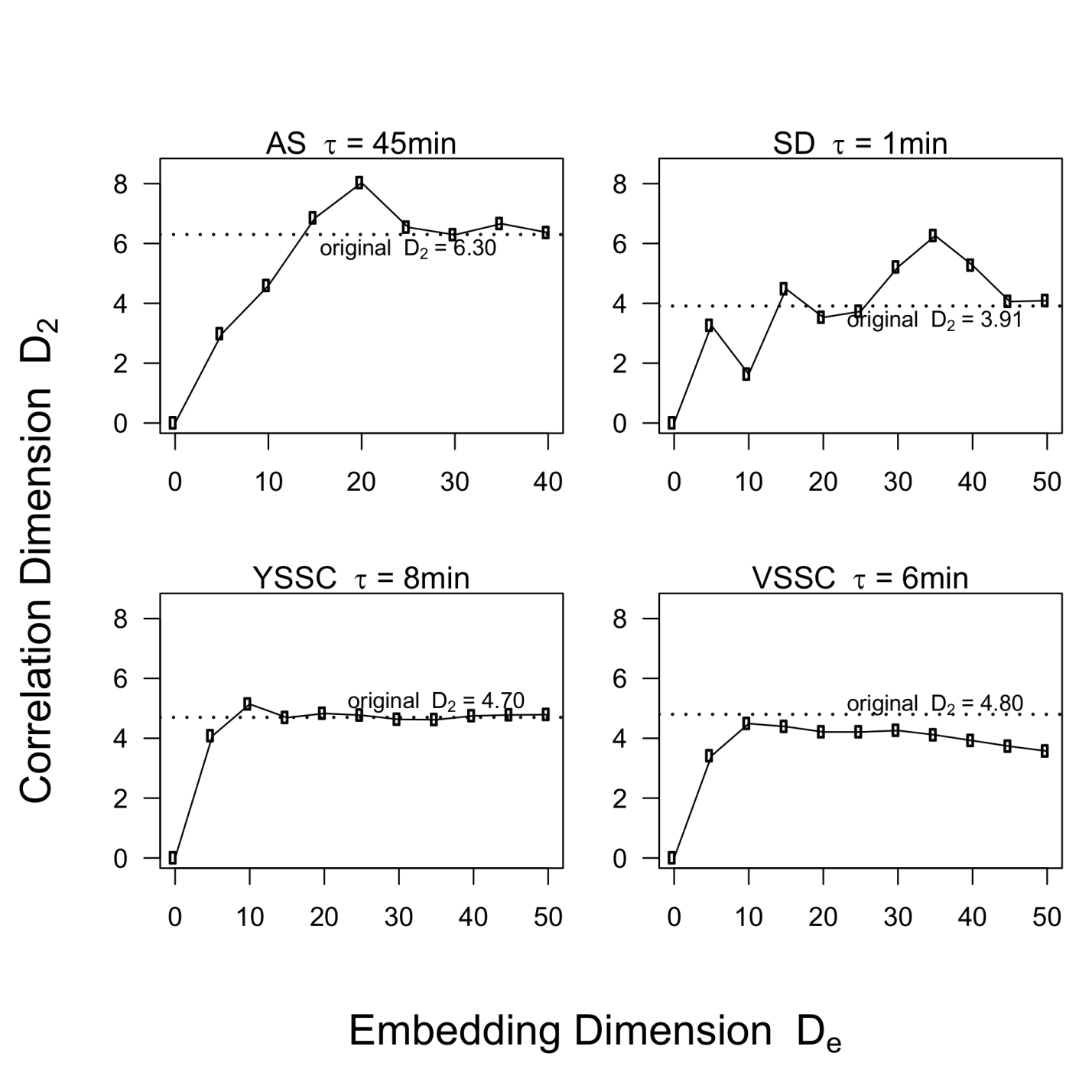


Figure S7: Takens’ delay embedding procedure: Reconstruction of the lower scale. Different time delays were used for different participants to construct the delay embedded vectors at each value of embedding dimension. As the embedding dimension is increased, the correlation dimension of the reconstructed delay embedded vectors asymptotes to a value close to the original lower scale dimension for each participant.

**Time delay selection**

Takens’ theorem guarantees a faithful reconstruction as long as the embedding dimension exceeds twice the topological dimension. However, Takens’ theorem assumes the availability of infinite noise-free data. In practice, the quality of the time delayed reconstruction using noisy and finite data is sensitive to the choice of the time delay τ. If τ is too small, there is very little gain in information across successive delay coordinates (*redundancy*, [17]) and the reconstructed trajectory is restricted to the main diagonal of the embedding space. If τ is too large, then successive delay coordinates may behave as though they were causally unrelated and this trajectory may not represent the true dynamics of the system (*irrelevance*, [17]). Additionally, in practical situations with finite data, the upper limit of possible τ’s is also constrained by the number of data points available in the time series.

There is currently no commonly accepted procedure for choosing τ that works equally well for all situations. Among the heuristics that have been developed, the most popular ones are based on autocorrelation and mutual information functions [18]. Autocorrelation measures the similarity between a signal and a delayed version of itself. Plotting the autocorrelation as a function of delay is expected to give us a measure of the transition from redundancy to irrelevance (discussed above). For example, τ can be chosen as the delay at which the autocorrelation function drops to 1/e of its initial value [19]. The autocorrelation method provides a satisfactory measure of dependence in linear low dimensional systems. While easy to compute, autocorrelation based heuristics for the choice of τ have several limitations. They are inconsistent; the same criterion of choice of τ based on the autocorrelation function does not work equally well for all dynamical systems (e.g. [19]) and they do not treat nonlinearity appropriately [18].

A popular class of heuristics that provides a better measure of dependency in nonlinear systems is based on mutual information [18]. Average mutual information (AMI) provides a measure of the predictability of a signal X(t+τ) given that X(t) has been observed. In order to reduce *redundancy* [17] between time delayed vectors constructed from a time series X(t), typically the first local minimum of AMI of the time series is chosen as the optimal τ when such a minimum is apparent.

The choice of τ is not an exact science and the criterion used to choose τ using AMI may also be inconsistent in determining optimal τ (e.g. [20]). The decline in AMI is related to the autocorrelation and in general, the shortest possible time delay that eliminates most autocorrelation is the recommended τ because longer the τ, more the data we lose in the embedding process. To illustrate this upper bound issue, let us consider NV’s data. There are 2181 data points in the time series. For an embedding dimension of 50 and a time delay τ = 40 indices (or data points, where the time difference between successive data points or indices = 1 min for NV’s data), we construct delay embedded 50 dimensional vectors in the following way: the first 50 dimensional time delayed (τ = 40) vector is [x_1_ x_41_ x_81_ x_121_ … x_1961_], the second vector is [x_2_ x_42_ x_82_ x_122_ … x_1962_] and the last possible delay embedded vector is [x_221_ x_261_ x_301_ x_341_ … x_2181_]. Note that the index of the 50^th^ element of the first vector is 1+(m-1)τ = 1+(50-1)40 = 1961. Therefore, we have already utilized 1961 of the 2181 available points for the first delay embedded vector for τ = 40 and m = 50. A total of 2181-1961+1 = 221 time delayed vectors are obtained. For our purposes, we need to calculate the correlation dimension of each set of delay embedded vectors. The correlation dimension is often used with small datasets because it considers O(N^2^) distances (where N is the number of points or vectors), and can therefore produce reliable estimates with fewer points.  Nevertheless, there is a practical lower limit on the number of points needed for a reliable estimate. The exact limit depends, of course, on the value of the dimensionality, but for the values considered here we know empirically that we need data close to the order of 10^3^ for a reliable estimate. Hence the availability of data points constrains the upper limit of τ that can be explored. If a higher τ must be used (based on the autocorrelation or the AMI plot), then there is an upper bound on the embedding dimensions that can be used (e.g. see Figure S7 for AS where we could only go up to 40 embedding dimensions).

Fortunately, many values of τ in the range suggested by the AMI plot work as shown in Figure S8 to reconstruct the correlation dimension. As long as the AMI has dropped sufficiently, we find that multiple values of τ yield faithful embeddings for higher embedding dimensions (as guaranteed for infinite noiseless data by Takens’ theorem and other extensions of it). In Figure S8, τ =10, 20 and 30 work approximately equally well for NV’s data. We presented τ = 10 in the manuscript since a lower τ gives us more delay embedded vectors for the correlation dimension calculation at each embedding dimension as discussed earlier. We repeated this procedure for each participant to determine the optimal time delay. τ_AS_ = 45 lags = 45 mins (since lag = 1 min for AS), τ_SD_ = 1 lag = 1 min (since we used an approximately equally spaced subset for SD’s data which was originally unevenly spaced, and the spacing of the subset is ~1min as explained in the previous section. We had to use all of the subset for the Takens procedure due to the unavailability of a sufficient number of points to try higher values of time delay), τ_VSSC_ = 45 lags = 6 mins (lag = 8 seconds for VSSC) and τ_YZSC_ = 60 lags = 8 mins (lag = 8 seconds for YZSC).


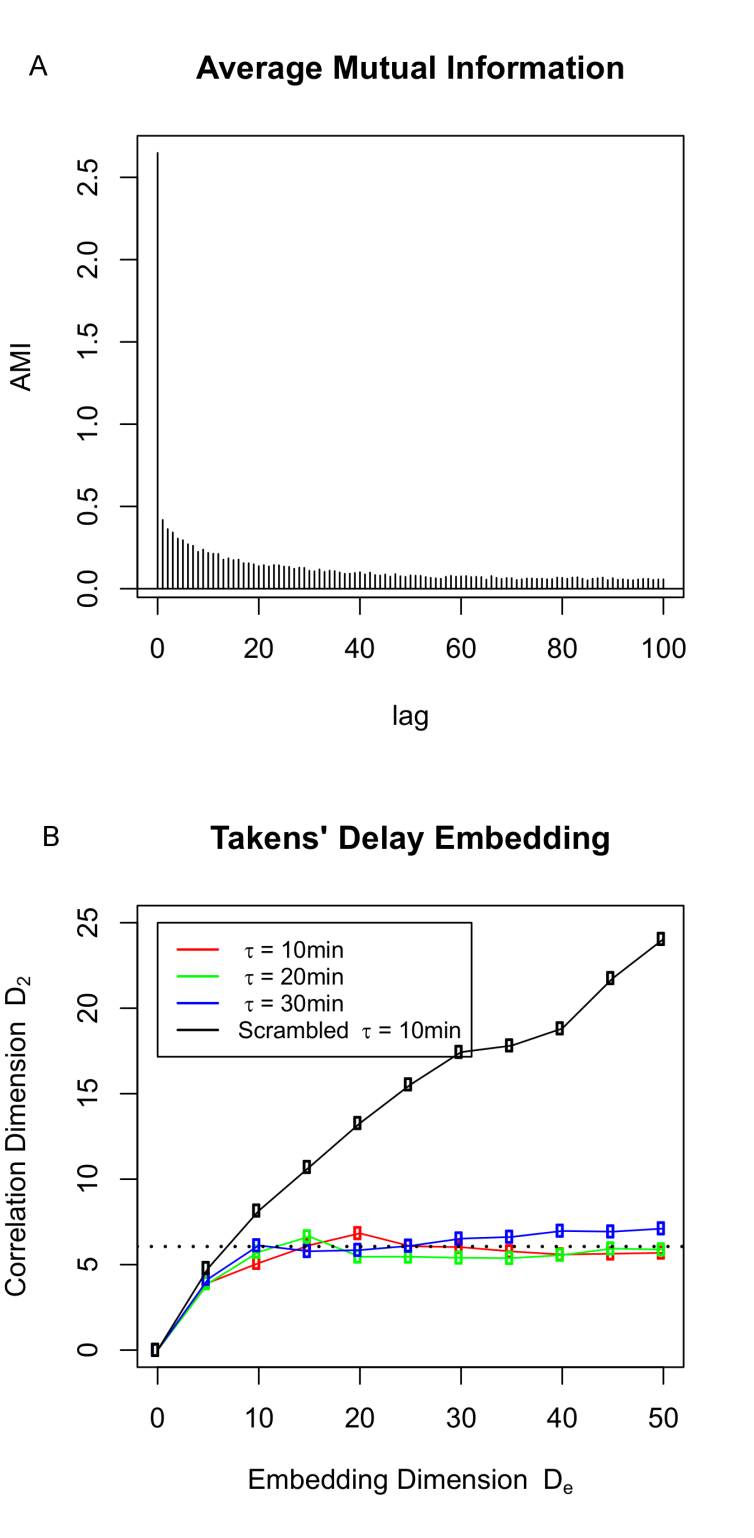


Figure S8: **A** The average mutual information plot of NV’s time series guides our search for an optimal time delay τ for Takens’ delay embedding procedure. **B** Takens’ embedding works approximately equally well for τ=10, 20, and 30 for NV’s data. In contrast, the randomized time series fills space, to within the limits of the number of points in the dataset, for all values of τ (only τ =10 is presented here for clarity).

**Supporting Information References**

1. Berry E, Conway M, Moulin C, Williams H, Hodges S, Williams L, Wood K, Smith G (2006) Stimulating episodic memory: Initial explorations using SenseCam. In Abstracts of the psychonomic society. 47th annual meeting 11, 56-57. Oxford University Press.
2. Swain M, Ballard D (1991) Color indexing. Int J Comput Vis 7: 11-32.
3. Huang J, Kumar SR, Mitra M, Zhu WJ, Zabih R (1997) Image indexing using color correlograms. In Proc IEEE Comput Soc Conf Comput Vis Pattern Recognit, 762-768.
4. Lowe DG (1999) Object recognition from local scale-invariant features. In Proc IEEE Int Conf Comput Vis 2: 1150-1157.
5. Lowe DG (2004) Distinctive image features from scale-invariant keypoints. Int J Comput Vis 60(2): 91-110.
6. Sreekumar V, Zhuang Y, Dennis S, Belkin M (2010). The dimensionality of episodic images. In R. Catrambone & S. Ohlsson (Eds.), Proceedings of the Thirty-Second Annual Meeting of the Cognitive Science Society. Austin, TX: Cognitive Science Society.
7. Smith, A. R. (1978). Color gamut transform pairs. Comput Graph (ACM) 12(3): 12-19.
8. Jeong S, Won CS, Gray RM (2004) Image retrieval using color histograms generated by Gauss mixture. Computer Vision and Image Understanding: Special Issue on Color for Image Indexing and Retrieval 94: 44-66.
9. Lindeberg T (1994) Scale-space theory: A basic tool for analysing structures at different scales. J Appl Stat 21(2): 224-270.
10. Doxas I, Dennis S, Oliver WL (2010). The dimensionality of discourse. Proc Natl Acad Sci USA 107(11): 4866-4871.
11. Chiu GS (2002) Bent-cable regression for assessing abruptness of change. Doctoral dissertation, Department of Statistics and Actuarial Science, Simon Fraser University.
12. Takens F (1981) Detecting strange attractors in turbulence. In: Rand DA, Young LS, editors. Lecture notes in mathematics. Berlin: Springer-Verlag. pp. 366-381.
13. Sauer T, Yorke J, Casdagli M (1991) Embedology. J Stat Phys 65: 579-616.
14. Whitney H (1936) Differentiable manifolds. Ann Math 37: 645-680.
15. Packard NH, Crutchfield JP, Farmer JD, Shaw RS (1980) Geometry from a time series. Phys Rev Lett 45(9): 712-716.
16. Grassberger P, Procaccia I (1983) Measuring the strangeness of strange attractors. Physica D 9: 189-208.
17. Casdagli M, Eubank S, Farmer JD, Gibson J (1991) State space reconstruction in the

presence of noise. Physica D 51: 52-98.

1. Fraser AM, Swinney HL (1986) Independent coordinates for strange attractors from mutual information. Phys Rev A 33: 1134-1140.
2. Albano AM, Muench J, Schwartz C, Mees AI and Rapp PE (1988). Singular-value

decomposition and the Grassberger-Procaccia algorithm. Phys Rev A 38: 3017-3026.

1. Martinerie JM, Albano AM, Mees AI and Rapp PE (1992). Mutual information, strange

attractors, and the optimal estimation of dimension. Phys Rev A 45: 7058-7064.
